# Investigating the effects of polymer plugging mechanism of liquid production decrease and improvement by the cross-linked gel performance

**DOI:** 10.1038/s41598-021-99385-8

**Published:** 2021-10-13

**Authors:** Kuiqian Ma, Mahamat Tahir Abdramane Mahamat Zene, Li Baozhen, Ruizhong Jiang, Haijun Fan, Yongzheng Cui, Liu Xiu Wei

**Affiliations:** 1 Tianjin Branch of CNOOC Co. Ltd, Tianjin, 300459 China; 2 State Key Laboratory of Offshore Oil Exploitation, Beijing, 100028 China; 3 China University of Petroleum (East China), Qingdao, 266580 Shandong China

**Keywords:** Geochemistry, Geophysics

## Abstract

Polymer flooding, as the most successful and well-known chemical EOR method was broadly applied around the world. Mostly, contrasted with Waterflooding, the production rate decrease during polymer flooding is smaller based on field application. Nevertheless, the production liquid rate decreased critically in the middle phase to late phase due to plugging, which could lead the way to poor flooding performance and fewer cumulative oil. In this work, first, we approached the affecting polymer plugging mechanism model on liquid production decrease to investigate the parameters such as; solid-phase concentration (SOLIDMIN), reacting frequency factor (FREQFAC) and others affecting components are all investigated consecutively. Secondly the model approached by cross-linked gel for the improvement of production liquid rate. The physical work was designed by a physical model, and then the polymer adsorption that generating blockage emerging in permeability diminish assessed by a mathematical model. The outcomes specify that the existence of this debris, excessive assemblage of solid-phase and the excessive reactant frequency factor has major mechanical and physical parameters effects on the reservoir throughout polymer flooding. Polymer flood model base case liquid ratio loss is 11.15 m^3^/day between the years 2014-08-01 to 2020-03-04. Comparing with the polymer flood model case 1, liquid ratio loss ranging to 1.97 m^3^/day between the years 2014-08-02 to 2020-03-03. While the oil ratio loss of the polymer flood base case model between the years 2015-07-08 to 2020-03-04 attained 12.4 m^3^/day contrasting with the polymer flood model case 1 oil ratio increase to 0.37 m^3^/day between the years 2014-08-04 to 2019-04-02. The cross-linked gel model base case liquid ratio loss is 2.09 m^3^/day between the years 2015-01-02 to 2020-02-03, while the oil ratio lost reached 9.15 m^3^/day between the years 2015-09-01 to 2020-02-03. Contrasting with the cross-linked gel model case 2 liquid ratio recovered from the loss and attained 25.43 m^3^/day in the year 2020-12-01, while the oil ratio is reached 15.22 m^3^/day in the year 2020-12-01. Polymer flood model examined through cross-linked gel model performed reliable outcomes by taking out the plugging, which also occasioned the reservoir production rate to decrease. With the application of cross-linked gel the affected parameters and the production rate have achieved an improvement.

## Introduction

Decrease in formation permeability or hydraulic conductivity has allured considerable discussion in the oilfield development period^1–3^. The dropping of permeability and mediocre hydraulic conductivity regularly lead the way to significant heterogeneity and low fluid production, also consequently impact oil production. It is frequently known as formation damage in an oilfield.

Particularly, composition of viscous emulsions can generate to bring about the misconception of pressure reaction viewed in polymer injectors (i.e. formation of oil bank, shear thickening effects) throughout well test analysis, mainly while projects encounter productivity losses^4^. Productivity loss in polymer floods constitutes an issue that has not been entirely approached in the literature excluding for less cases that will be shortly outlined afterward in this part. Utmost of polymer floods observation from (1978–2016) outlined untimely in this research did not announce the reduction of production rates (total fluids) in the projects evaluated^5^. Detailed that low productivity is one of the problem that are confronting polymer flood field applications.

But, this examination nor presented detailed information of potential roots of productivity losses neither specified field projects to back this affirmation^6^. Outlined uttermost pertinent challenges and matters described in polymer floods (i.e. corrosion, emulsion formation and mechanical collapse) together with a representation of six disappointing situations^7^. This evaluation, nevertheless, did announce productivity losses undergone in Bohai Bay project in China^8^. Productivity losses in polymer floods have been assigned to numerous factors and its feasible amalgamation:

- Skin damage nearby the wellbore (i.e. asphaltene deposition, mud formation, etc.)
- Plugging outcomes because of the existence of solid fine fragments
- Decreased fluid removal in artificial lift pump structure because of expansions in fluid density in the existence of produced polymer
- Stable oil–water–polymer emulsions

In general, solid-phase invasion, fines migration, scale formation, as well as water sensitivity^8–12^ can occasion pollution and plugging nearby the wellbore; therefore has an unfavourable impact on oil production.

Acidizing treatment is one of the extremely crucial techniques that used in aiming to bring out the pollution and recover formation permeability^12–16^.

Normal synthetic polymers are partially hydrolyzed polyacrylamide (HPAM) also its derivatives.

HPAM have been utilized for large-scale production in numerous fields due to it is lower cost^17^.

Additional polyacrylamide-derived polymers applied in EOR involving hydrophobically associating polymers, salinity tolerant polyacrylamide (KYPAM), as well as 2-acrylamide 2-methyl propane sulfonate (AMPS)^18^.

Some fields illustrated the implementation of biopolymers like xanthan gum, including cellulose for EOR^19,20^. Biopolymer not just reduces the water to oil mobility ratio still as well particularly plugs the high-permeability thief zones also lead the way to a reorientation of the water-flood to inaccessible oil zones^21^.

Laboratory outcome indicated that this particular plugging is because of the gesture of resident bacteria, whichever can nourish unless on hydrocarbons instead on biopolymer like xanthan gum^21,22^.

In addition, polymer gels, polymer-enhanced foams, plus foamed gels has been additionally applied to ameliorate oil recovery alongside plugging the high permeability thief zones^23^.

Water production in oil-producing wells becomes increasingly significant as the wells mature, in addition to it is extremely crucial to control water production also enhance oil recovery^23–26^. Out of numerous profile control methods, in-depth profile control techniques have been progressed rapidly plus performed favourably, including cross-linked polymer microspheres^25,27^. Moreover, retard crosslinking polymer gel which is greatly applied due to its higher gel stability also acceptable plugging effect^27–30^. Some of the two foremost concerns that require more observation in the usage of procrastinated crosslinking polymer gel systems; the gelation time as well as the gel stability.

The gelation time controls the length of gel penetration while the polymer gel system injected toward formation layers, whereas the salt resistance, as well as temperature resistance achievement of the polymer, regulate the gel solidity of the gels system. Modifiable gelation time is essential in procrastinated crosslinking polymer gels systems throughout the profile control operation^28–32^.

There are several methods to check the gelation time also, the key point is to check the liberated rate of the cross-linkers. The more frequently applied polymer gel systems in profile control are hydrolyzed polyacrylamide (HPAM) as well as chromium (III) ions^33^.

Gels arranged with metallic cross-linkers like chromium, aluminum, along with zirconium, have lessened stability toward rising temperature with short gelation duration, and also are not beneficial in rising temperature reservoirs profound profile control operation. The crosslinking ions can be initiated into chelated ions to raise the gelation duration, but there are restraints to the gelation plan because of the insufficiency command over the liberate kinetics of chelated crosslinking ions. One more well-known water-based gel system for water-control utilizations was a phenol/formaldehyde cross-linker system^30,32–35^. The phenol/formaldehyde polymer gel system is thermally steady; besides, the gelation duration is manageable over a vast temperature scope^30^. Various emulsions can moreover was used to raise the gelation duration for procrastinating the crosslinking polymer gel systems^36,37^.

The more frequently utilized polymer in the polymer gel systems for water shut-off is Hydrolyzed polyacrylamide (HPAM). The viscosity improvement of HPAM is most likely up till the augmentation of solvated chains, due to the repulsion of carboxylate groups. HPAM indicates mediocre salt resistance, temperature resistance, as well as shear resistance performance, which delayed its implementation in high temperature as well as high salinity reservoirs^37–41^.

As an outcome, numerous polymers were expanded also used in high temperature along with high salinity reservoirs, like modified xanthan, amphiphilic polymer like hydrophobically modified polyacrylamides (HMPAM), also more. For instance, the modified xanthan/chromium gels are resistant to no less than 120 °C^42^.

Contrasted with HPAM, HMPAM indicates a lot better salt resistance, temperature resistance, also shear resistance accomplishment; exceedingly higher thickening property; also good emulsification performance, moreover, consequently can expand each of the swept volume as well as displacement efficiency^42–52^.

In the Cartesian coordinate dimension of the well block enlarge, velocity damage, moreover, consequently shear rate as well as in consequence polymer viscosity is inaccurately counted^53,54^.

This physical adsorption about polymer remains simulated alongside Langmuir isotherm calculation. Polymer adsorption conceivably diminishes this effective permeability^55^.

This is kind of permeability diminution is calculated alongside a permeability diminution factor (Rk) who defined to a proportion with effective permeability toward brine as well as polymer solution. This outcome of permeability diminution remains undertaken to be irreparable also carry on afterwards polymer flooding as well as is named residual resistance factor (RRF). The RRF interpreted like the proportion of this mobility with a brine solution ahead also afterwards this polymer injection. The diminution of the permeability factor is likely considerable despite many polymers like Xanthan gum, or during this establishment, permeability occur higher^56^.

The injectivity reduces because of the permeability diminution element terminate; meanwhile, polymer retention achieves to the greatest extent.

Throughout this activity of water plugging, mostly this frequent non-success circumstance alike polymer gel simply is infiltrated by the ensuing water passage because of this failure of the polymer substance. Consequently, recently developed water passage established is also the extension about this swept away capacity happened to be restricted^57^. These, due to plugging the fracture, need a polymer gel alongside a steady scheme along with an inflated mechanical solidity, who can entirely bung this water passage also preserve an acceptable achievement under the prolonged cleaning of the injected water.

In conformance with the numbers of Changqing oilfield and Yanchang oilfield, these mostly used polymer gels are the gel fragments with the in-situ cross-linking polymer gel. Gel particles have a concentrated three-dimensional mesh shape alongside a vigorous energy storage size, an elevated solidity, also a high viscoelasticity^58^.

The objective of this research is to investigate the main parameters causing the plugging mechanism on the reservoir production rate. Along with applying cross-linked gel for improving the affected parameters and boosting the production rate.

The affecting parameters were assessed through CMG STARS software. The indicated analysis introduces a clear method for the forthcoming study.

The higher proceeding simulators STARS is entirely presented thermal compositional model (Rubin and Buchanan, Oballa et al.) such utilized earlier within a wide variety of primary recovery besides secondary recovery simulation to many years. Additionally, as typical reservoir gridding capacities, entirely integrated well models, together with forward numerical solution approach plus adjustable implied algorithms, its adaptability in explaining many fluid flow methods with structure is especially significant. Such last level capabilities comprise the outcomes of proceeding supplements as follow; polymer, gels, surfactants, including foams^59^.

Therefore, this frequent multi-element non-equilibrium originally build for in-situ combustion modeling used in order to illustrate supplementary non-equilibrium combining in addition to condition substituting operation like foamy oils^60^. The usefulness of this sort of capability in the modeling of sand migration besides production will be enlarged in this work.

## Methods

**Figure 1:**
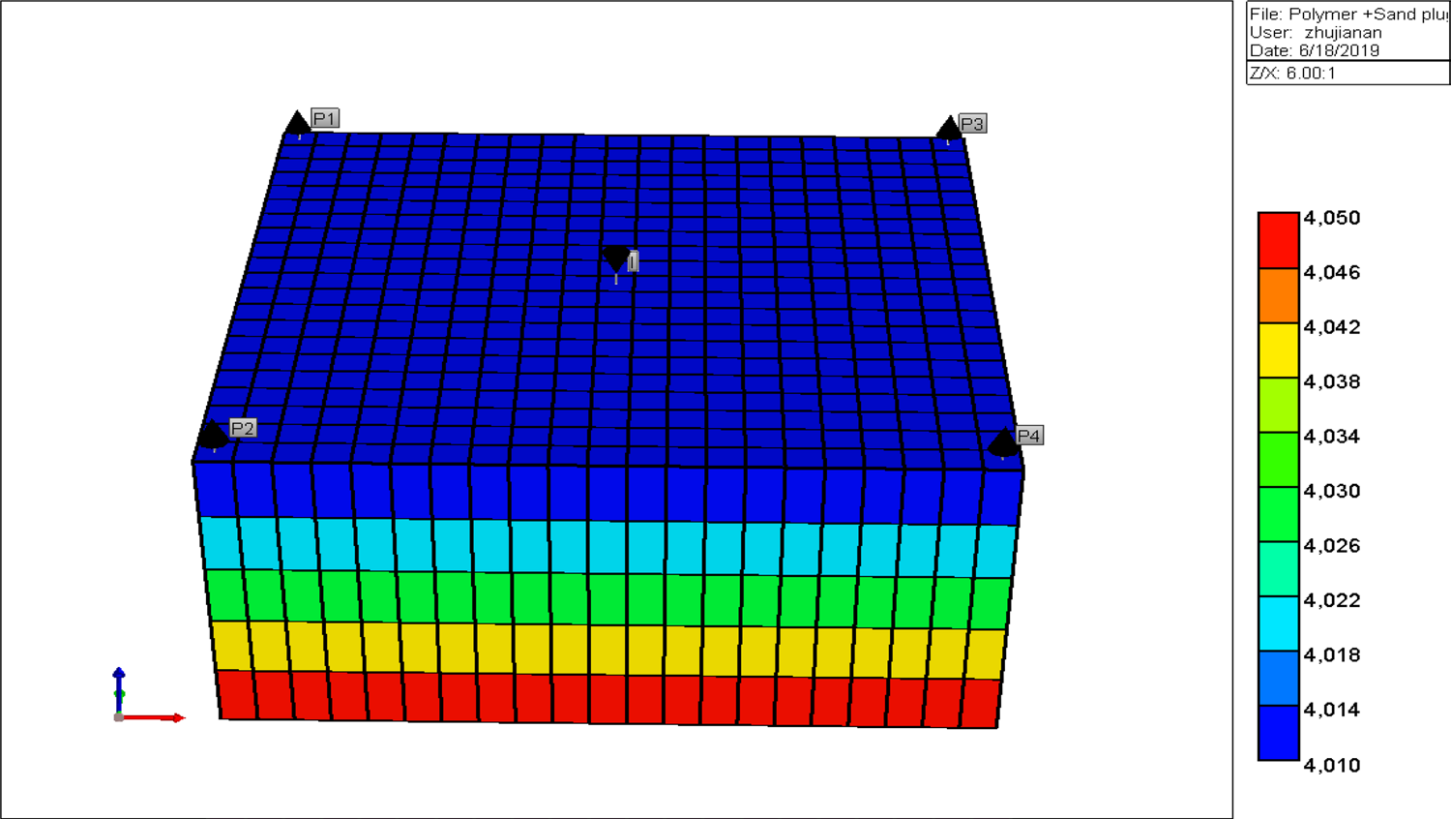
3D model of inverted five (5) spot pattern polymer flooding model *CMG STARS 2017.10* URL: https://www.cmgl.ca/.

This research work was formed on a five spot-pattern model of 21 × 21 × 5 grid blocks in total 2205 are active was been modelled to accomplish this analysis. X, Y with a mesh size of 40 ft. It was partitioned toward five layers in distinction to uppermost orientate toward the base. Such explain the median thickness of the primary layer is 5 ft. Water Oil Contact (WOC) about 4050 m moreover the Gas Oil Contact (GOC) about 4000 m. This permeability differs through primary toward the 5th layer (X, Y, as well as Z), orientation like presented in Table 1, and (see Fig. 1)^61,62^.

**Table 1:** Base case model description and physical properties of the test scenarios.

Parameter description	Units	Values
Cell in X direction (DX)	X grid block sizes, ft	21
Cell in Y direction (DY)	Y grid block sizes, ft	21
Cell in Z direction (DZ)	Z grid block sizes, ft	5
Total cell number	Total cell of the block, ft	2205
Porosity	Grid block porosity values, %	0.23
Permeability in X direction	Permeability in X direction, mD	500, 1000, 1600, 1050, 1700
Permeability in Y direction	Permeability in Y direction, mD	500, 1000, 1600, 1050, 1700
Permeability in Z direction	Permeability in Z direction, mD	50, 100, 160, 105, 170
Water mobility weight	Water MW kg/gmole	18.02
Polymer	Polymer MW kg/gmole	8
Dead oil	Dead oil MW kg/gmole	1
Sand	Sand MW kg/gmole	1.25
N_moving sand	Nonmoving sand MW	1.25
Reference pressure	Reference pressure, kPa	18,000
Water density	Water density kg/m^3^	1061.99
Polymer	Polymer density kg/m^3^	794.4
Dead oil	Dead oil kg/m^3^	954.999
Sand	Sand density kg/m^3^	2650
N_moving sand	N_moving sand kg/m^3^	2650
Water liquid compressibility	Water liquid compressibility, kPa	3.3e−6
Polymer liquid compressibility	liquid compressibility, kPa	3.3e−6
Dead oil liquid compressibility	liquid compressibility, kPa	8.96e−6
Sand liquid compressibility	liquid compressibility, kPa	1e−007
N_moving sand liquid compressibility	liquid compressibility, kPa	1e−007
Water compressibility	Water compressibility, Psia	0.000725189
Polymer injection	Wt% PV	1.75
Dead pore volume	Dead Pore, PV	0.15
Residual resistance factor	Residual Resistance Factor (RRF)	1.5
Initial water saturation	Initial water saturation, %	0.5
Reference temperature	Temperature, C	60
Connate water saturation	Connate water saturation, %	0.2

The reservoir water injection well is specified upon restraint STW surface water ratio of 100 m^3^/day, alongside a BHP controlled at 17,000 kPa on utmost. The producer wells are specified to function on BHP (bottom hole pressure) of 16,000 kPa. The liquid phase viscosity table, and a lower range of $25$ °C to an ultimate range of $100$ °C.

The non-equilibrium blockage mechanism operated with the SOLIDMIN and the FREQFACT with differing ranges for the base cases (polymer flood model and cross-linked gel model) beside the sensitivity cases in Table 2.

**Table 2:** List of test cases for the established study of the polymer model and cross-linked gel model.

Scenario	Initial solid concentration%	FREQFACT
Base case	0.0052	0.031
Base case	0.0052	0.031
Case 1	0.023	0.002312
Case 2	0.023	0.000002

**Figure 2:**
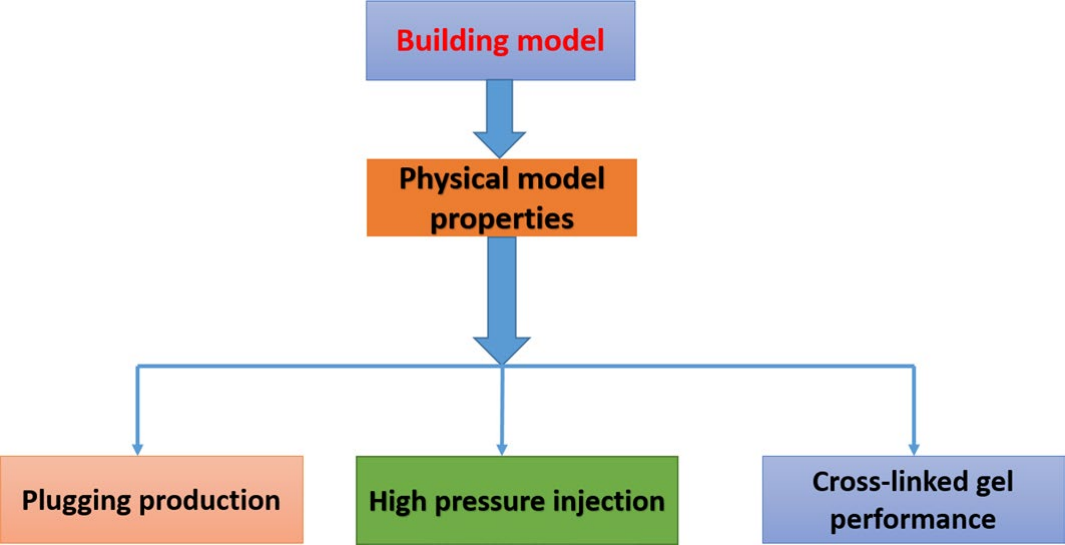
Workflow and software usage *CMG STARS 2017.10* URL: https://www.cmgl.ca/.

**Figure 3:**
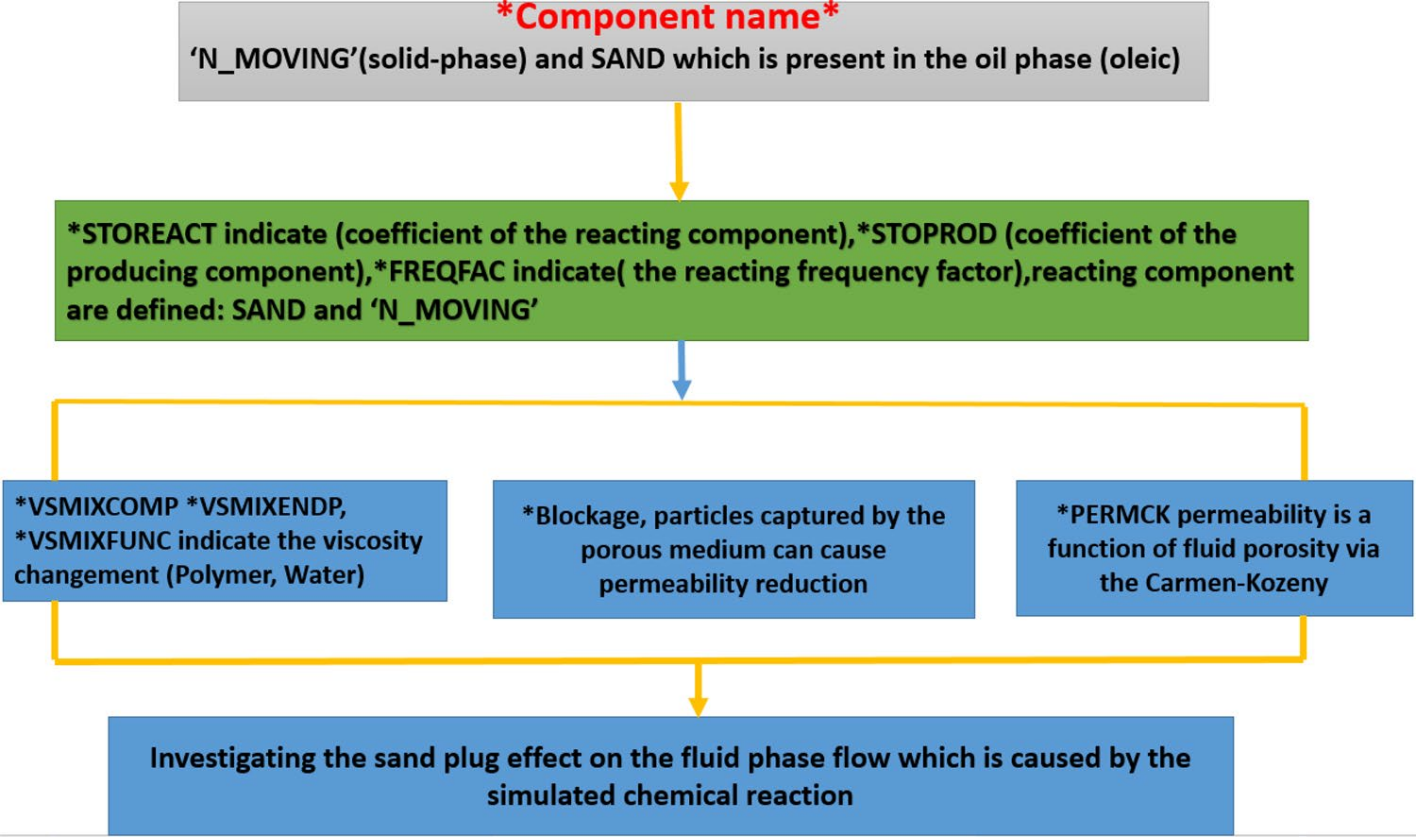
Keyword description of the Polymer sand plug model *CMG STARS 2017.10* URL: https://www.cmgl.ca/.

**Figure 4:**
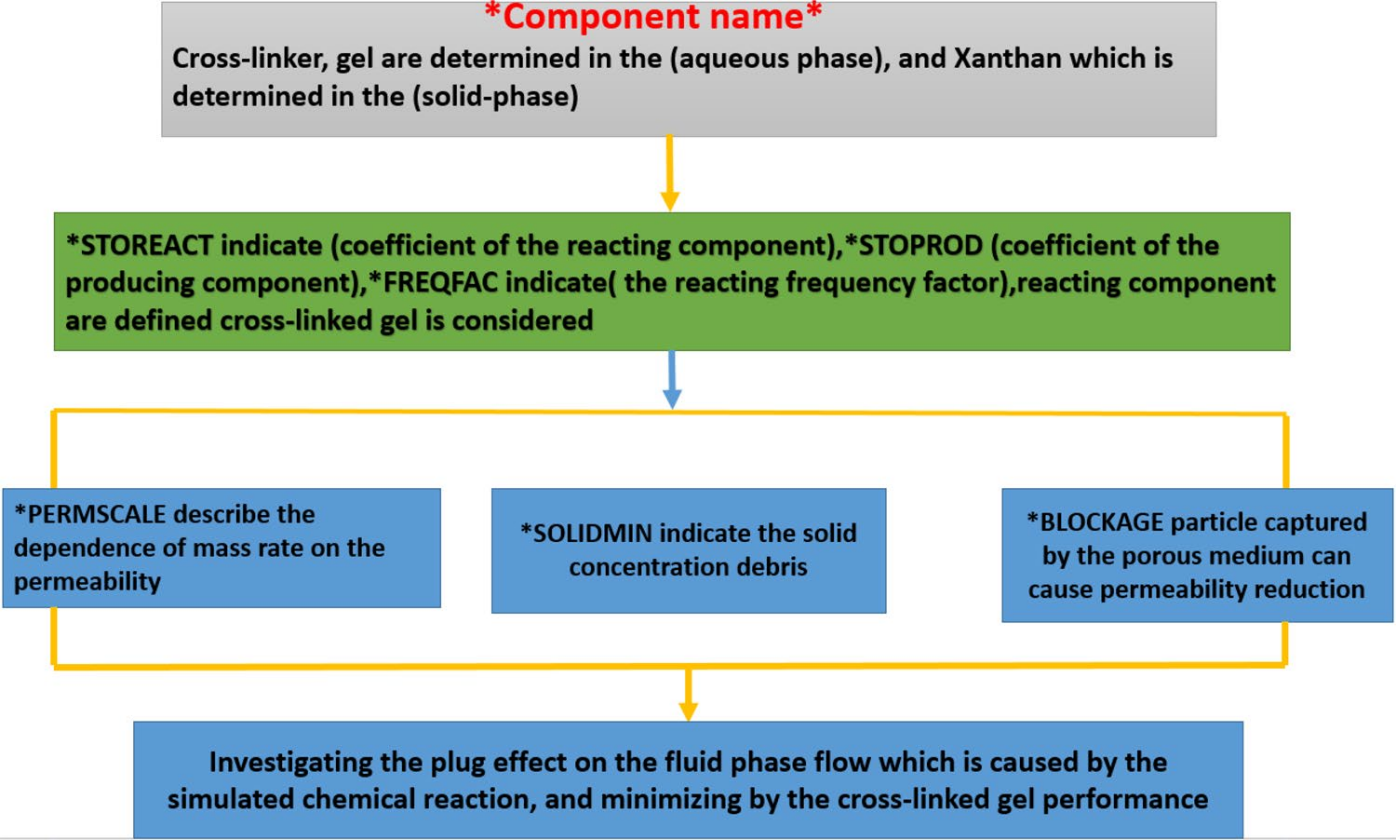
Keywords description of the cross-linked gel reaction model *CMG STARS 2017.10* URL: https://www.cmgl.ca/.

For the achievement of this work, CMG STARS software was used^63^ as described in more details in Fig. 2 Workflow and software usage considered, then the key components with descriptions models of Figs. 3 and 4.

During the application of polymer flooding model, the sand migration along with movement treatment were approached as follow in this work:

'N_MOVING': Sand composition which appear in solid-phase which remains non-moving or stagnant (Solid). Stagnant sand possibly causes permeability decrease (blockage) in particular to equilibrium mass transfer to the rock (Adsorption).

SAND: A sand composition which is available toward oil stage (oleic), who brought within oil moreover liberated whereas the entrance of the operating formation. The presence of this element possibly varies oil viscosity within material affecting the migration of sand about the producer well surrounding.

('N_MOVING'): The Initial solid concentration which is fixed with a concentration of 0.052 along with the oil mole fraction (sand) of 0.0025 determined; each one is evaluated by the software.

(STOREACT): The influence of reaction frequency activation energy that appear toward the reaction constituent of CMG STARS (STARS USER GUIDE), shows the frequency factor of the reacting portion. Such contributes a crucial part to the outcome, additionally the plugging influences. Through model ingredient, it was recognized such chemical element positioned through the stoichiometric proportion, every accumulation specified about, it is mass throughout the sand plug model (polymer, Sand, 'N_MOVING') reaction outcome in sand-solid consistency.

By specifying the foremost polymer molecular mass of the polymer, it is advantageous for appraising reservoir permeability as well oil viscosity.

This gel is predominantly utilized throughout this worldwide due to its adherence, alongside injecting the concentration of gel, lessen the permeability of elevated areas which occur obstructed or plugged regardless of influencing this little permeable area. It explains that additional oil were recuperated out of the non-swept fewer permeable areas. This fundamental modeling method goes along with^64^.

The fluid input model was detailed alongside six chemical elements as follows: water, polymer (polyacrylamide), cross-linker (phenol–formaldehyde) also gel established into the aqueous phase while dead oil, as well as solution gas were determined into the oleic phase, the polymer, along with gel elements as well adsorbed through the rock.

Polymers will be classified as biopolymers (natural polymers) and synthetic (plastic) polymers.

Natural polymers were produced by microscopic organisms such as bacteria, fungus whereas synthetic polymers were synthesized by scientists (or humans)^65^.

By injecting polymer toward the formation, encircled within the inactive proportion. Beneath a number of the succeeding circumstances: high temperature, and high pressure, the plugging alongside complicated mechanisms remains initiated.

In order to barrage or plugging issue eradication, a model input constituted of the subsequent chemical elements: Water, polymer, cross-linker, and gel altogether set into the aqueous phase, xanthan into (solid-phase) as well as dead oil into (Oleic phase).

Polymer, gel as well as xanthan elements altogether soaked up within this rock. Polymer, xanthan, as well as cross-linked gel are entirely used for: porosity, permeability, water viscosity, polymer deterioration, inaccessible pore volume, the elevated accumulation of solid-phase also the elevated reactant frequency factor.

Xanthan can resist to mechanical degradation (shear), and is thermally steady between the temperature limit of $70$–$90$ °C^66^.

However, these composite is especially sensitive to bacterial deterioration once injected toward this field consisting of lower temperature areas in the reservoir. In addition, it has already been outlined that xanthan can transport a number of cellular junk that result in plug^67^.

In this current research work, some of the following polymers were considered; Hydrolyzed Polyacrylamide (HPAM), biopolymers (xanthan) and synthetic polymer as a gel.

HPAM is a polyelectrolyte that interconnect firmly alongside ions in solution. The adaptability of HPAM polymeric interconnected structure form further interactive to the ionic solidity of aqueous solutions and therefore additional sensible to salt/hardness contrasted to additional polymers like xanthan gum^68^.

Xanthan gum is a biopolymer liberated alongside the microorganism Xanthomonas campestris as well is produced economically at the fermentation operation. Aqueous solutions of xanthan gum are extremely viscous overdue to the existing dual with triple helix structure of the polymer chain accompanied by enormous polar side bonds that forward considerable hydrogen bonds^69^.

Gel injection is one of the most efficient techniques of EOR to increase oil recovery. Water management can be a better option than increasing oil production^70,71^. Especially in heterogeneous reservoirs, gel injection, could be selected instead of polymer injection^72^.

The purpose of applying gels for oil recuperation has begun during the 1990s. Biopolymers and synthetic polymers which form gelation, thickening or emulsification/stabilizing agents have been used successfully in the oil reservoirs^48^. With the commercially available biopolymers and synthetic polymers in aqueous solutions, this mixture produces gels as a result of the thickening in water^73^. The movable gels have high viscosity that leads increase of oil mobility. Gels have been used in highly permeable reservoirs to reduce permeability. Hydrogels or water-based gels decrease the permeability of water rather than oil permeability^74^.

Similarly, oil-based gels reduce the permeability of oil compared to water permeability. Resistance factor increases due to high viscosity of water and low permeability that shows an increment of recovered oil^75^.

Microgels, performed particle gel (PPG), pH-sensitive cross-linked polymers are the commercially available gels to use in the petroleum reservoirs^76^.

The interaction between gels and water can make the gels shrink, swell or stay in the same size based on the gel type, the salinity of water, temperature and shear rate^77^. PPG are strength and size-controlled, environment friendly, and their stability is not sensitive to reservoirs minerals and formation water salinity. The size of PPG can be controlled based on the pore sizes to decrease permeability. Gels have been applied to heterogeneous reservoirs to form the reservoirs homogenous^78^. Therefore, gels injection may also be preferred for the reason of its enhancement of sweep efficiency prior to injecting a costly polymer.

The blockage mechanism is used for the distinct cases of the polymer model and the gel cross-linked are investigated under the identical model.

The permeability reduction is on account of mechanical entrapment beside it demonstrated as phase permeability decrease^79^.

The permeability influencing or varying while polymer flooding and cross-linked gel model cannot be observed in the three-dimensional model outcome.

The permeability decrease is relying on the adsorption, input elements alongside ADSCOMP. The rock depending on adsorption is detailed as shown:

These replacements prior to polymer flooding also afterwards polymer flooding possibly evaluated through the Eq. (1) below;

1 $$ \overline{K} = \frac{{K_{bp} }}{{R_{K} }}. $$

The adsorption element is determined like the adsorption element, residual resistance factor, and desorption proportion depends upon this permeability of the formation.

Reservoir heterogeneities generate those properties to vary significantly throughout the bounds of a reservoir. Appropriately, equilibrium adsorption remains a justification of position in addition to component concentration as well as temperature.

The adsorption can generate the clog up who is the reduced amount in the effective permeability. Such is considered as for ascending the adsorption acquired out of delimited concentration along with temperature circumstances alongside the factor^80^.

2 $$ \begin{aligned} & R_{KW} = \frac{1 + (RRF - 1) \times AD(C,T)}{{ADMAXT}} \\ & R_{Ko} = \frac{1 + (RRF - 1) \times AD(C,T)}{{ADMAXT}}. \\ \end{aligned} $$

While the amount of adsorption degree rise through time variation, $R_{k}$ vary starting with 1.0 to a very higher $RRF$^81^. Ahead of the utilization of polymer flooding, the RRF alongside mobility differentiation can be formulated like specified by;

3 $$ RRF = \frac{{\lambda_{Wb} }}{{\lambda_{Wa} }} = \frac{{K_{wb} }}{{K_{wa} }}\frac{{\mu_{wb} }}{{\mu_{wa} }}. $$

Polymer flooding lessens the permeability to a particular layer, predominantly provided this solid accumulation stage of the polymer adsorption, who in consideration of reducing the permeability of all layers.

A linear fluid flow by Darcy law intended to remain used in that manner; because of non-variant flow rate and entire pressure reduce is $\Delta_{p}$ the entire of the diminish of every single layer.

4 $$ K_{{}} = \frac{{Q_{{}} \times L \times \mu_{{}} }}{{A \times \Delta_{p} }}, $$

where; $$\Delta_{p} = \Delta_{p1} + \Delta_{p2} + \Delta_{p3} \ldots \Delta_{pn}$$.

Displacing the pressure decrease by Darcy equation for each of the five (5) layers as well as permeability;

5 $$ \frac{{q\mu La_{{}} }}{{Ak_{a} }} = \frac{{q\mu La_{1} }}{{Ak_{1} }} + \frac{{q\mu La_{2} }}{{Ak_{2} }} + \frac{{q\mu La_{3} }}{{Ak_{3} }} + \frac{{q\mu La_{4} }}{{Ak_{4} }} + \frac{{q\mu La_{5} }}{{Ak_{5} }}. $$

Concluding the equivalent designation then defining the equation will appear as:

6 $$ K_{a} = \frac{Le}{{\left( \frac{La}{k} \right)1 + \left( \frac{La}{k} \right)2 + \left( \frac{La}{k} \right)3 + \left( \frac{La}{k} \right)4 + \left( \frac{La}{k} \right)5}}, $$

7 $$ K_{a} = \frac{{\sum\nolimits_{e = 1}^{n} {L_{e} } }}{{\sum\nolimits_{e = 1}^{n} {\left( {L_{a} /k} \right)_{e}^{{}} } }}, $$

where; AK determined as usual grid block permeability for water as $AK_{w} (i,j,k)$ an issue and for oil as $AK_{o} (i,j,k)$. The relative mobility of the phase carrying an absorbing component was certainly influenced by the viscosity and blockage.

Where; $(i,j,k)$ indicate the grid block permeability flow in a different directions.

The permeability reduction influences the permeability of water and oil were presented as listed below; $AK_{w} (i,j,k),AK_{o} (i,j,k)$ as;

8 $$ AK_{w} (I) = \frac{{AK(i,j,k) \times K_{RW} }}{{R_{KW} (i,j,k)}}, $$

9 $$ AK_{o} (i,j,k) = \frac{{AK(i,j,k) \times K_{RO} }}{{R_{KO} (i,j,k)}}. $$

Polymer adsorption who cause in permeability decrease evaluated alongside the factor as shown;

10 $$ R_{K} = \frac{{K_{w} }}{{K_{po} }}. $$

The non-accessible pore volume (IPV) primary was recommended by (Daws on and Lauthz 1972), a certain number of pore were not attained while the polymer flooding process moreover it was in the end acknowledged by (Pancharoen Thiele and Kovscek 2010), this effective polymer porosity was no more compared to the realistic. The below pore volume method will be fulfilled toward the aqueous polymer solutions by;

11 $$ \phi_{p} = (1 - IPV) \times \phi . $$

## Results and discussion

### Test cases and justification

#### Base case: polymer sand plug model

Sand plugging arising examined pointing to the production well; 1D model in addition differentiated along with the graph figures while was introduced in the outcomes.

A few years ago, a blockage took place that was depicted in one-dimensional (1D) model numbers in such a way that it affected the fluid rate and amount of oil rate in the reservoir while taking production into account.

Sand plugs arise from the investigation of production points in a 1D model, in addition to the differentiation of graph numbers and the introduction of results.

**Figure 5:**
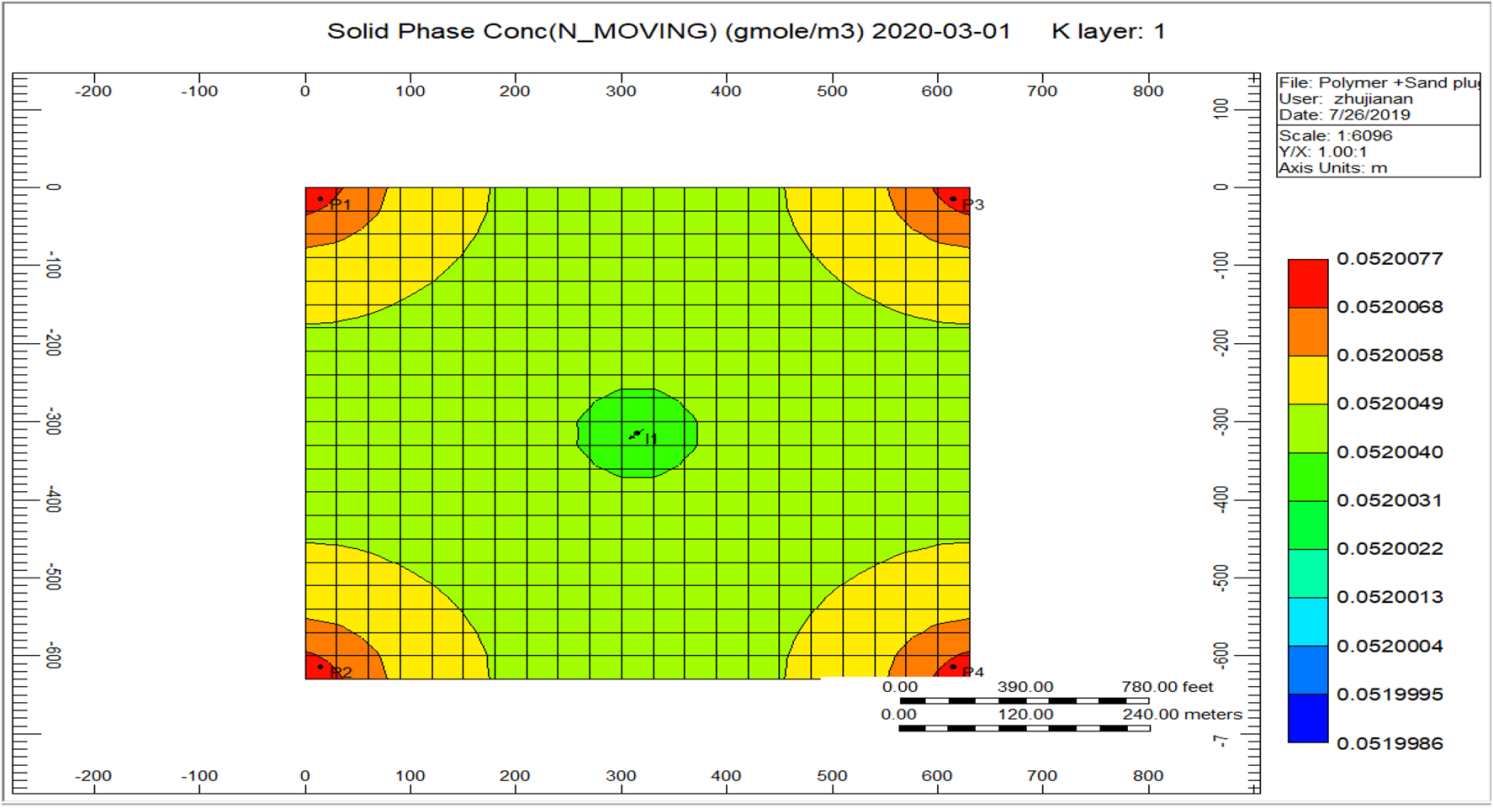
Indicates the presence of a concentration of sand nearby the production well *CMG STARS 2017.10* URL: https://www.cmgl.ca/.

Figure 5 indicates the presence of a concentration of sand during production, in the range of 0.0520077, and the solids concentration decrease with the injector well from 0.0520031 to 0.000031. Sand plug model and distinct color scale indicate the occurrence of sand in the production direction, which reaches a considerate extent over time from a small area.

**Figure 6:**
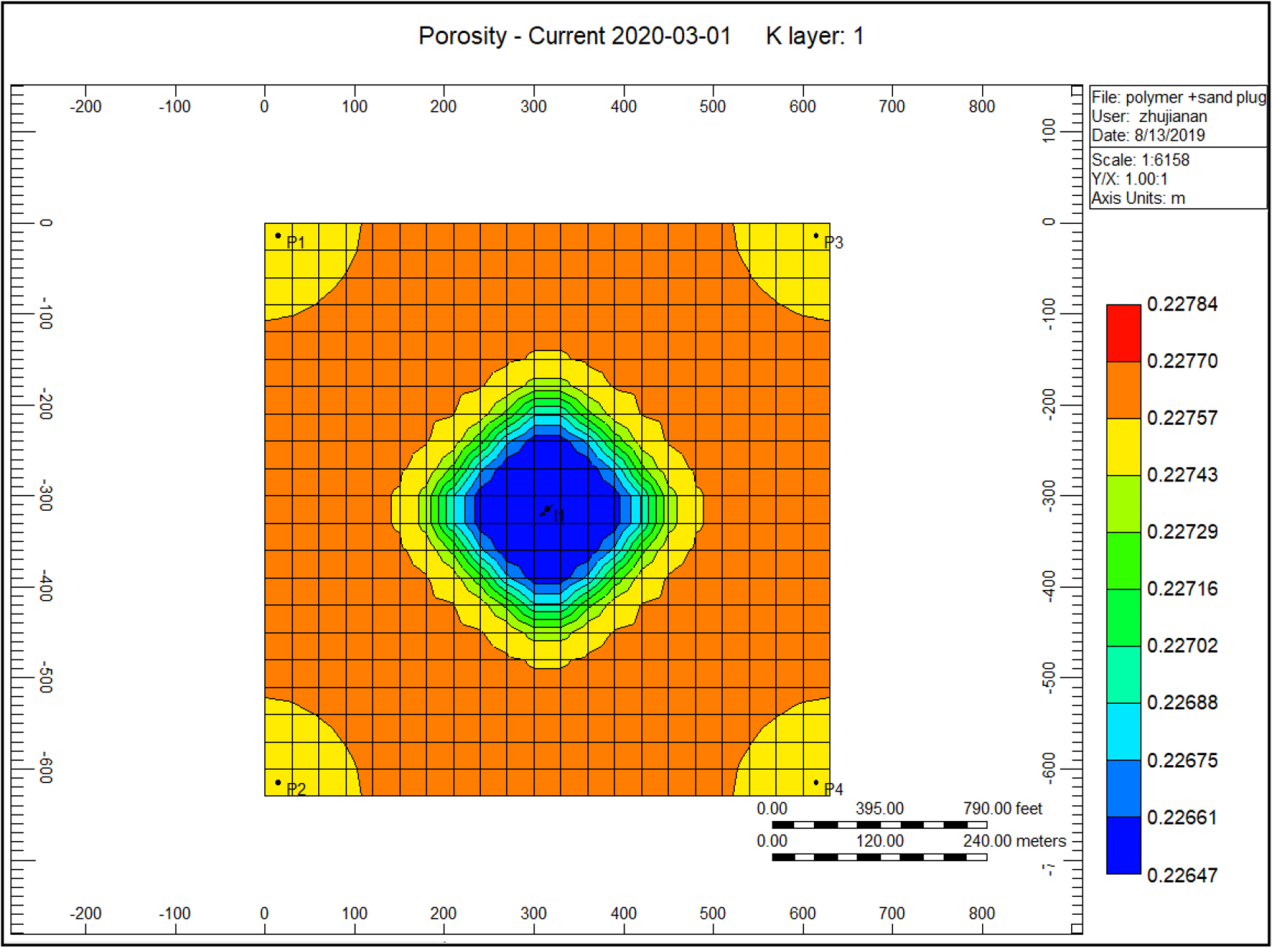
Shows the porosity reduction through the polymer sand plug model *CMG STARS 2017.10* URL: https://www.cmgl.ca/.

Figure 6 shows the porosity reduction through the polymer sand plug model; the yellow porosity diminishes sighted at the location of producer well in the range of 0.22743. Nevertheless, in the midway, model nearby the injector well porosity lessen with a lower range of 0.22647. That is due to the presence of mechanical entrapment, debris provoking porosity to decrease.

**Figure 7:**
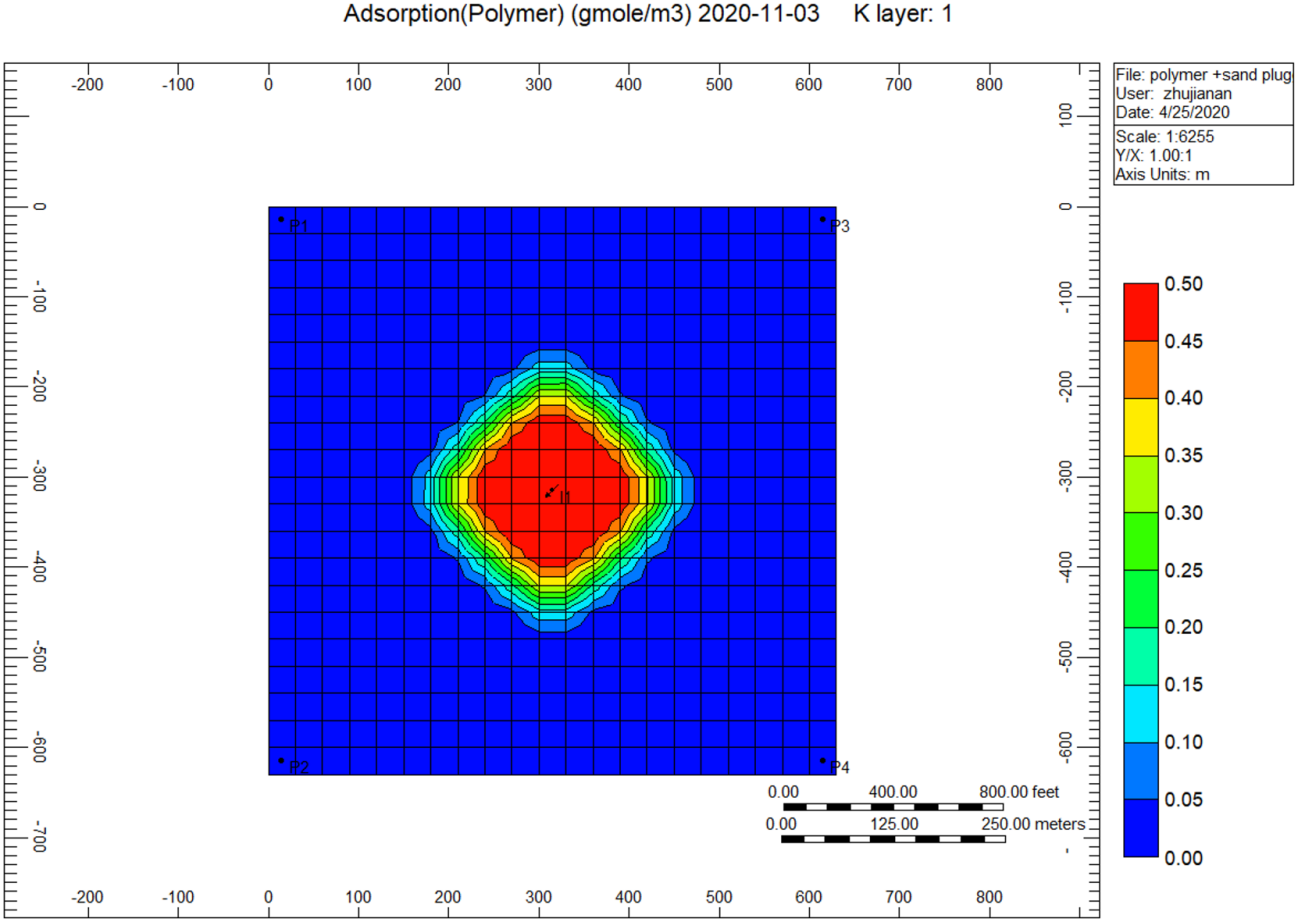
Shows the polymer adsorption throughout the polymer sand plug model *CMG STARS 2017.10* URL: https://www.cmgl.ca/.

Figure 7 shows the polymer adsorption throughout the polymer sand plug model, we can remark the appearance of high adsorption of polymer bordering the injector well alongside a portion of 0.50, whereas a range of 0.01–0.00 surrounding the producer well.

**Figure 8:**
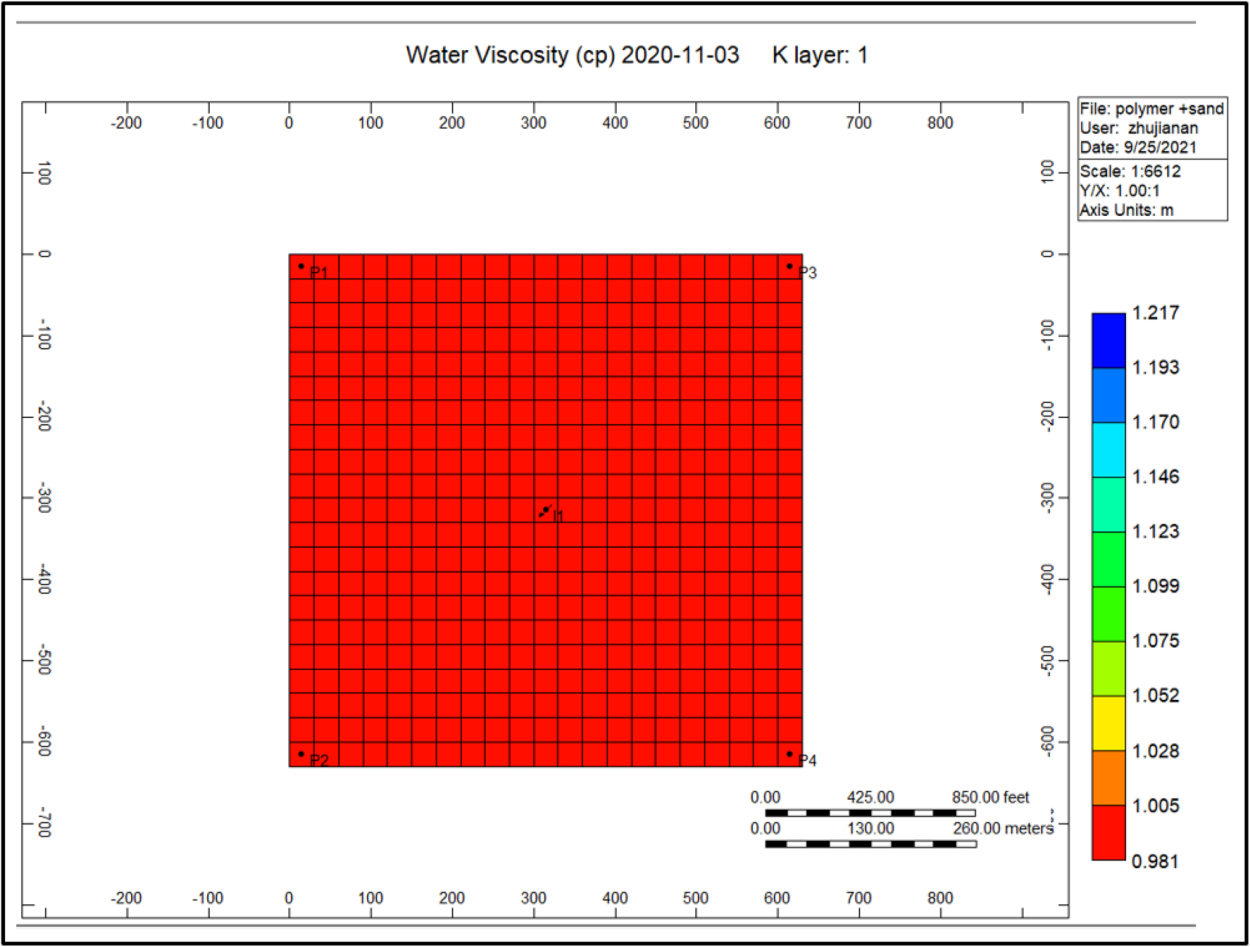
Shows the water viscosity through the polymer sand plug model *CMG STARS 2017.10* URL: https://www.cmgl.ca/.

Figure 8 shows the water viscosity through the polymer sand plug model, in addition to the polymer deterioration, this viscosity decreased to it is lowest throughout the injection of polymer flooding. The viscosity ratio diminution reached to a high rise of 0.981.

The steady rate lessens because of the high-level existence of sand-solid concentration component reaction of non-moving ('N_MOVING') including the moving SAND whichever existing in the oil affecting through the polymer flooding. Such a high-level reduction in addition on account of high sand-solid concentration portion of (0.052), together with the FREQFACT range of 0.031 influence the liquid including the oil rate to decline endlessly.

Based on our outcomes, we find that the variables influencing the liquid ratio and oil ratio to decrease on account of the existence of sand fragments and scrap resulting from the clogging of polymer floods. As shown: polymer adsorption, polymer degradation, diminution of permeability, decreased porosity, inaccessible pore volume, water viscosity volume, the elevated solid-phase accumulation plus a high reactant frequency factor, accompanying by an elevated proportion of impact on the liquid rate and oil rate, which reduce the derived parameters of reactant frequency factor as well solid-phase accumulation and additionally cause low production.

**Figure 9:**
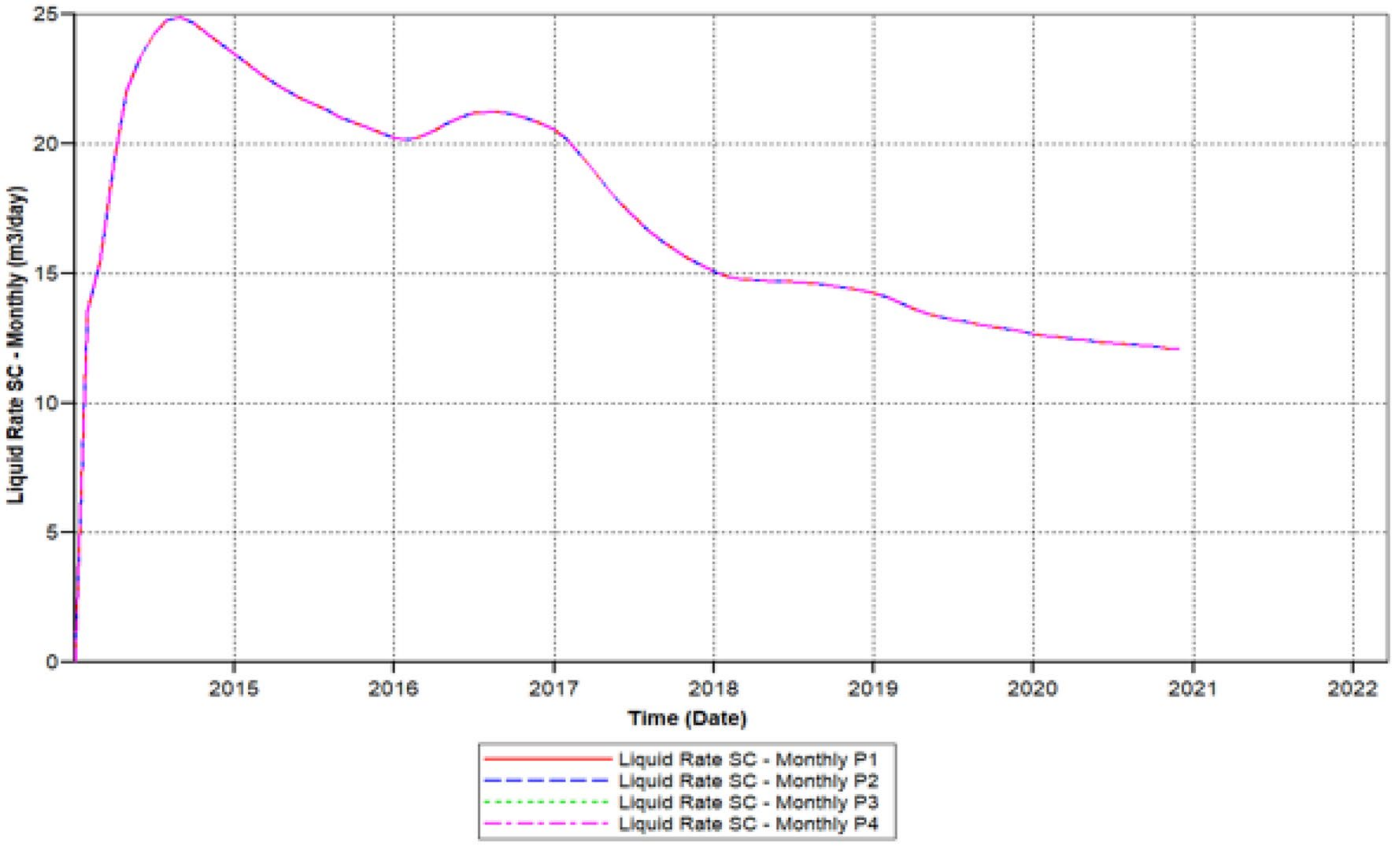
Shows the polymer sand plug model liquid ratio *CMG STARS 2017.10* URL: https://www.cmgl.ca/.

Figure 9 shows the polymer sand plug model liquid ratio attained to it is elevated ratio of 25 m^3^/day within 2014-08-01 steadily declining up to it has outreached a ratio about 14.68 m^3^/day in 2019-05-02, then this ratio continued decreasing before it has attained to this a lower rate of 13.85 m^3^/day in the middle of 2020-03-1.

During continuous production, the liquid ratio declined, in the following years (2014-09-02 and 2015-07-09) in relation to 1.410 m^3^/day, which could not be regenerated. Over time, the oil production ratio decreased from 2015-07-09 and 2016-06-02, the production rate fell rather than increased, with only a portion of 3.2 m^3^/day not recovered. In contrast, in the three of the four years from 2016-06-02 to 2020-03-04, the drop is remarkable, a complete ratio of 10.4 m^3^/day lost. At the same time, the production oil ratio decreased from year 2017-02-02 to 2018-03-04, with a decrease proportion of 4.66 m^3^/day. Eventually, in the year 2018-03-04 as to 2020-03-04, the loss is smaller than earlier years alongside oil ratio non-recoverable about 2.37 m^3^day.

**Figure 10:**
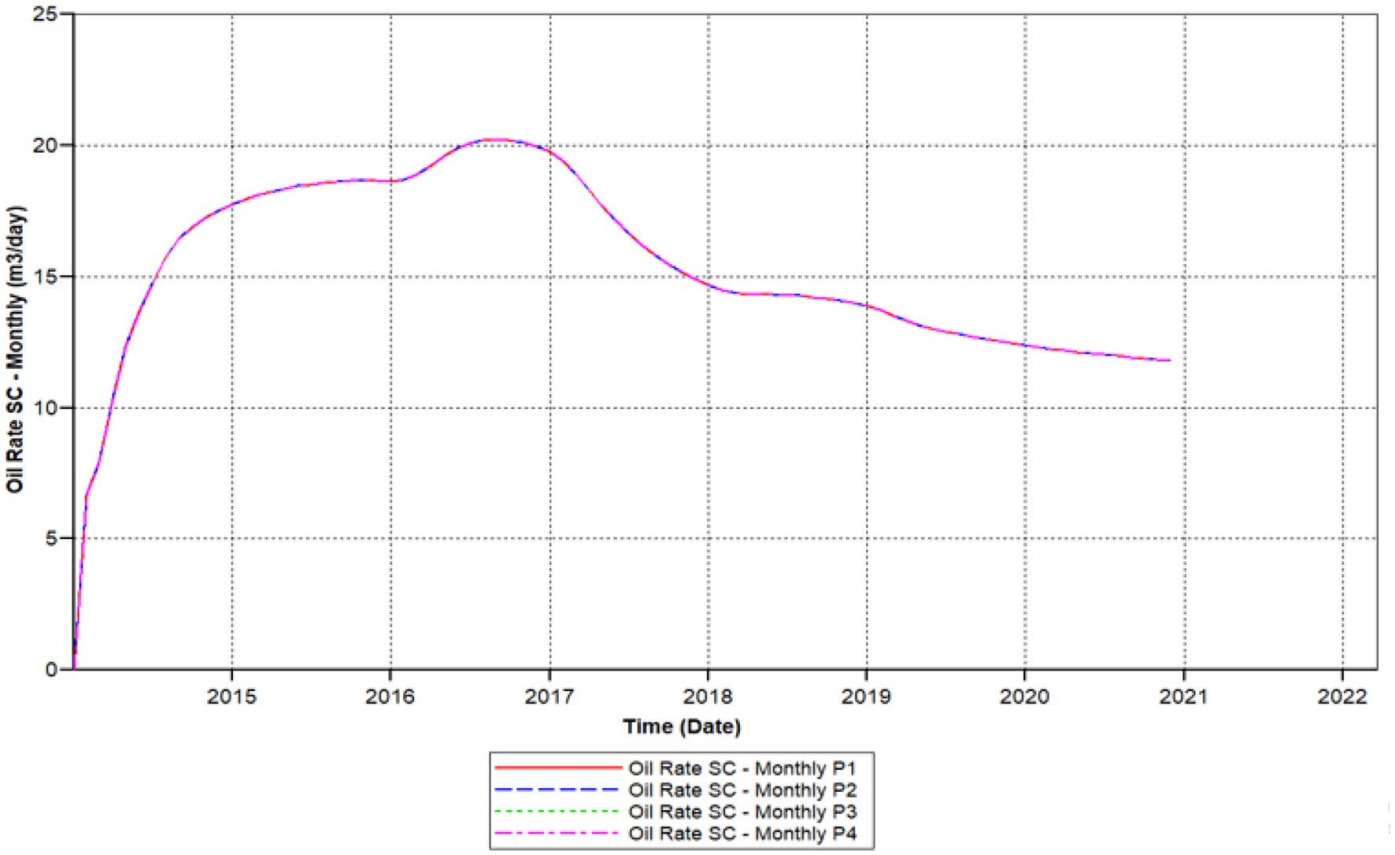
Shows the polymer sand plug model oil ratio *CMG STARS 2017.10* URL: https://www.cmgl.ca/.

Figure 10 shows the polymer sand plug model oil ratio, the ratio is continuously increasing attained 20.1 m^3^/day in 2015-07-08 but started decreasing from 2016-02-02 up to it has outreached toward a beneath ratio of 12.56 m^3^/day within 2020-03-04.

#### Base case: cross-linked gel model

Since sand plugging circumstance taking place while the polymer flooding is influencing the liquid ratio together with the oil ratio to decrease endlessly, exploring for an obstructive treatment to remove polymer plugging and ameliorating the liquid ratio as well as the oil ratio is extremely vital.

The polymer sand plug model alongside these corresponding variables examined throughout cross-linked gel accomplishment for this purpose of diminishing necessarily plugging impact. Accompanied by the implementation of a cross-linked gel method, those affected variables such as; cross-linked gel adsorption, permeability, porosity, water viscosity, the accumulation of solid-phase decreased, and this liquid production rate has been improved after the removal of plugging.

Gao-Qian-Bei block located within Ji-Dong field, Shengli encountered an active edge aquifer water, a cross-linked polymer used to increase the oil recuperation^82^.

**Figure 11:**
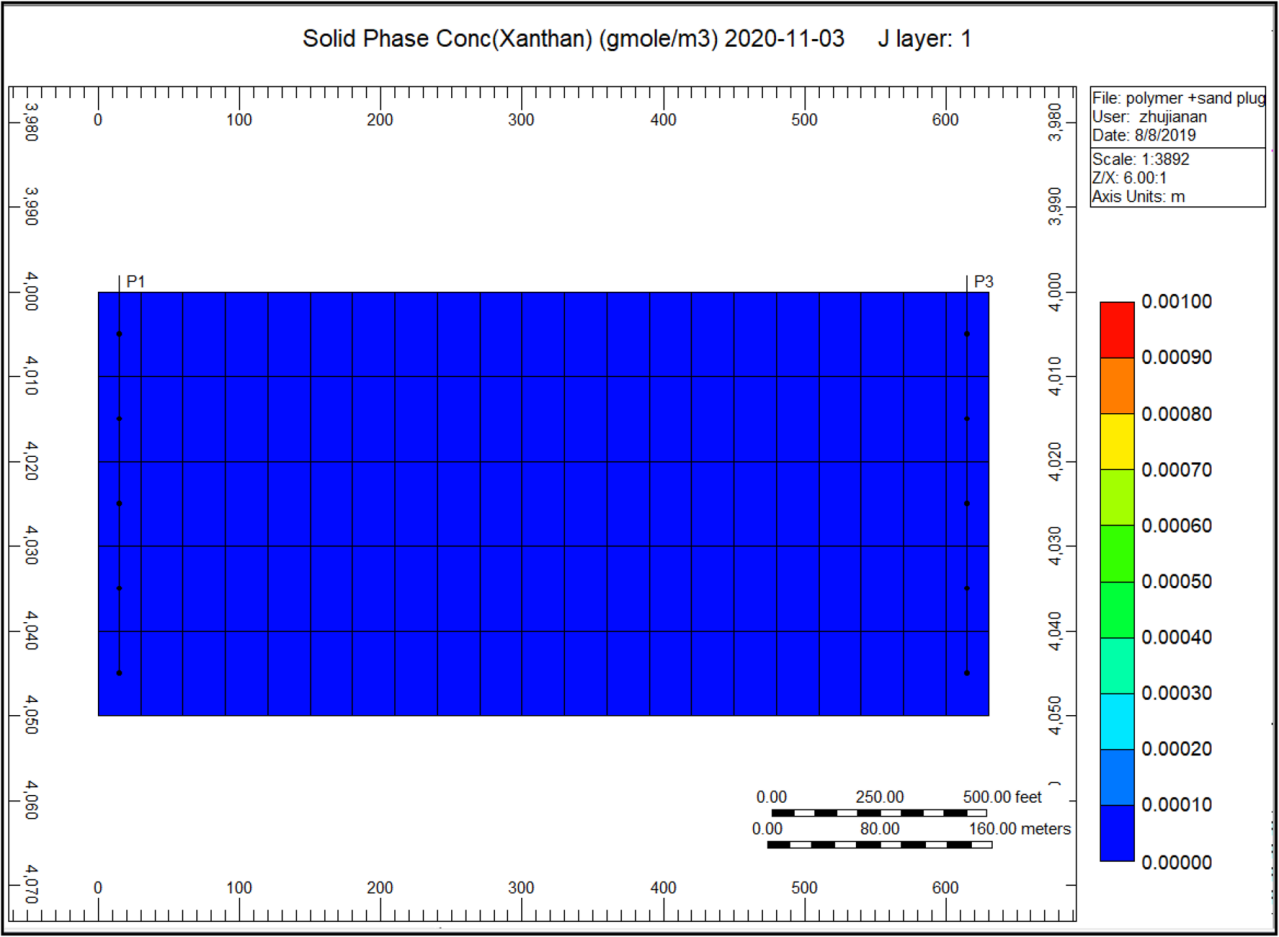
Indicates the cross-linked gel solid-phase concentration in the direction of the production well *CMG STARS 2017.10* URL: https://www.cmgl.ca/.

Gel conformance treatment is widely used around the world for the reason of its effectiveness. Reviewed by Seright, this initial deliberation in order to identify the foremost variation uniting a gel applied for reservoir conformance control in addition to a polymer flood. Conventional gels utilized in “conformance constraint” are considered to obstruct instead decrease the circulation volume of elevated permeability conduits with no sabotaging the fewer permeable hydrocarbon profitable section. It is consequently mandatory to reduce the inflow of gelants toward this not so much as permeable^83^. Figure 11 Indicates the Cross-linked gel solid-phase concentration in the direction of the production well; throughout last year, we can observe that the accumulation of solid-phase varies from 0.000120 to 0.00 compare to Fig. 5 Indicates risen accumulation of sand nearby the production well, this solid-phase concentration was highly gathered surrounding the model. With the application of cross-linked gel, the solid debris concentration will be reduced.

**Figure 12:**
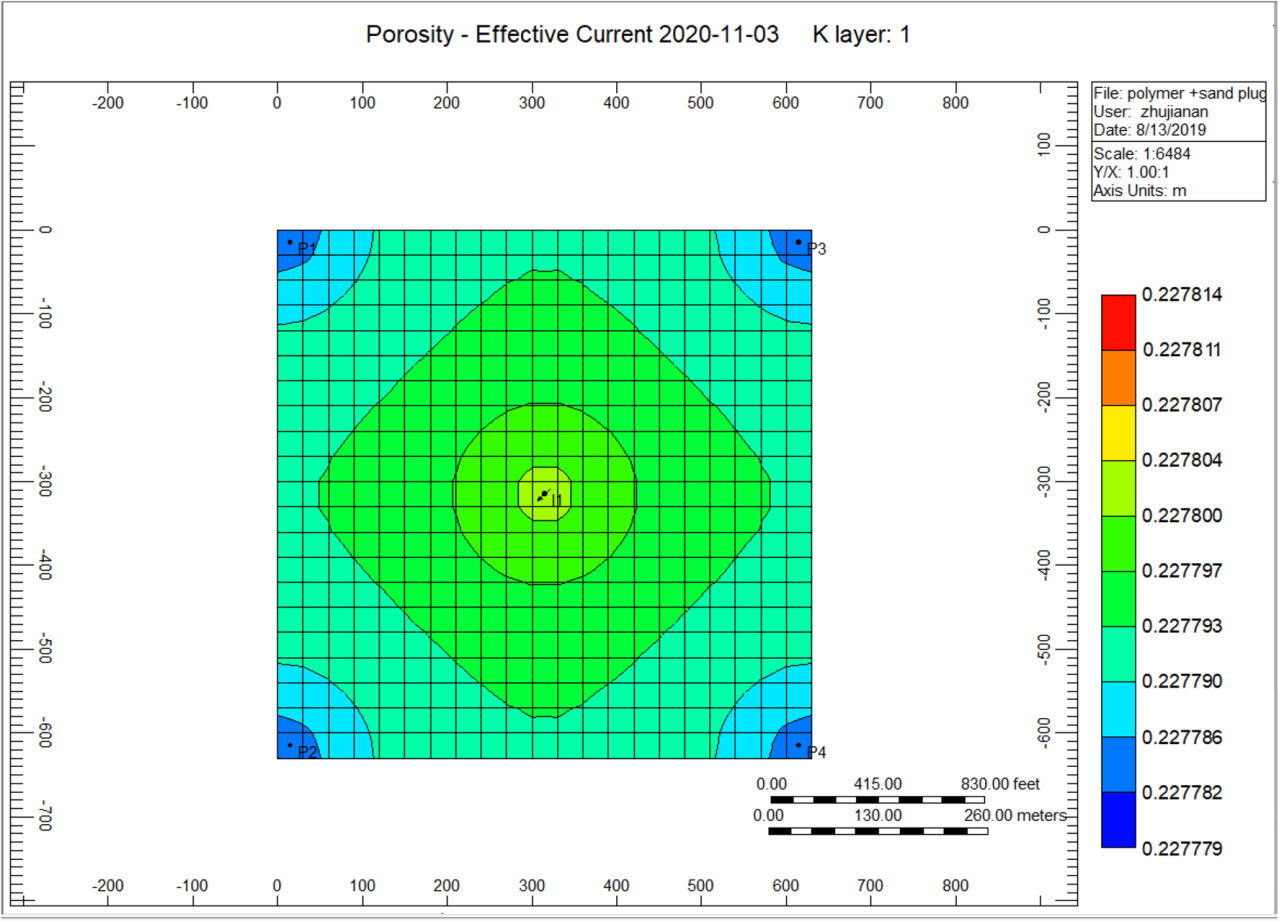
Indicates the porosity increase of the cross-linked gel performance model *CMG STARS 2017.10* URL: https://www.cmgl.ca/.

Figure 12 indicates the porosity rise of the cross-linked gel performance model, the porosity at the place of an injection well alongside an increasing portion of 0.2277800. However, at the position of the producer well decreased to a range of 0.227782. Comparing to Fig. 6 shows the porosity reduction during the polymer sand plug model, the porosity was improved.

**Figure 13:**
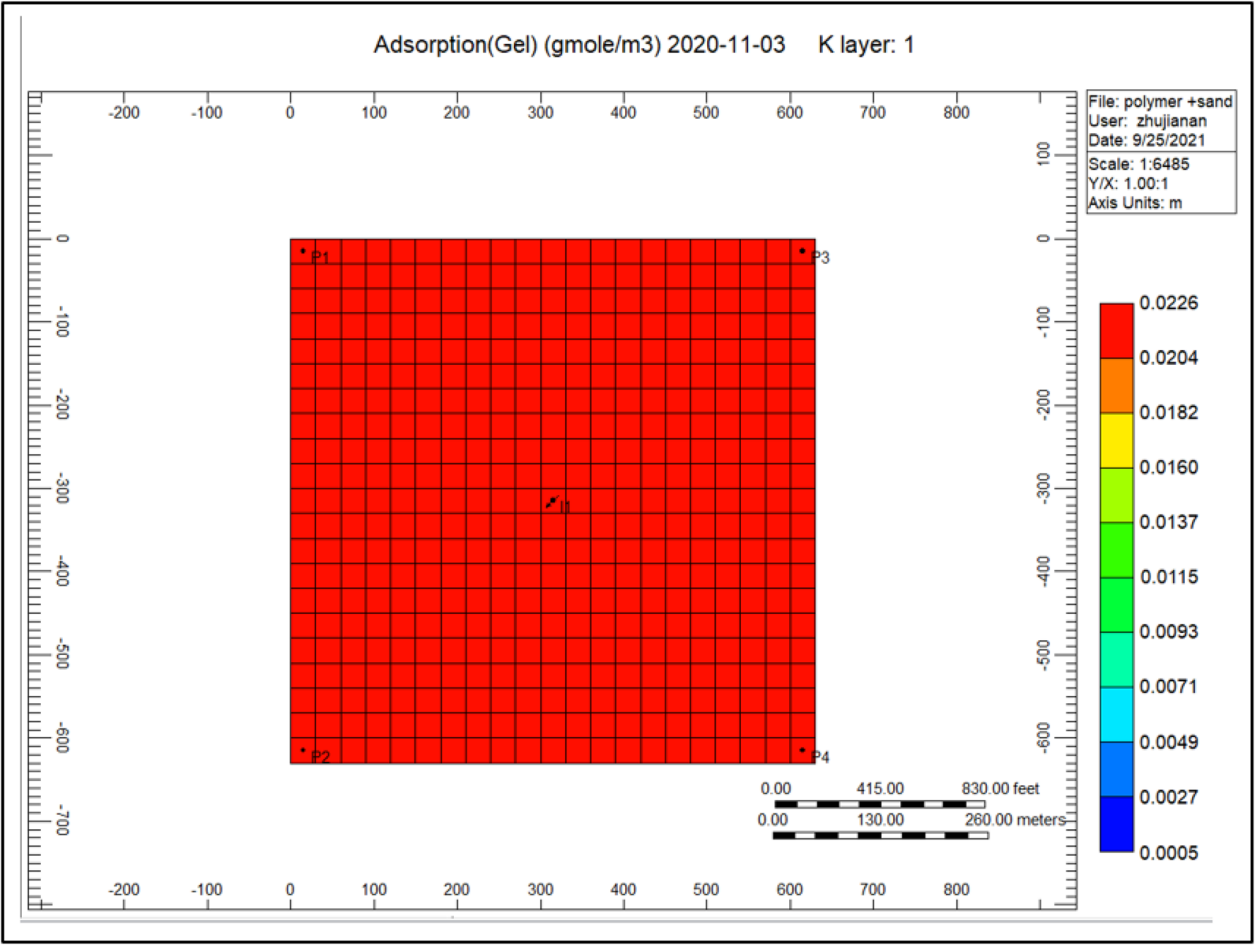
Indicates the gel adsorption of the cross-linked gel achievement model *CMG STARS 2017.10* URL: https://www.cmgl.ca/.

Figure 13 gel adsorption of the cross-linked gel achievement model, we can notice from the previous year that cross-linked gel is exceedingly soaked up alongside a range of 0.022601 within the complete model comparing to Fig. 7 shows the polymer adsorption throughout polymer sand plug model, this absorption is hugely concentrated toward the injector well.

**Figure 14:**
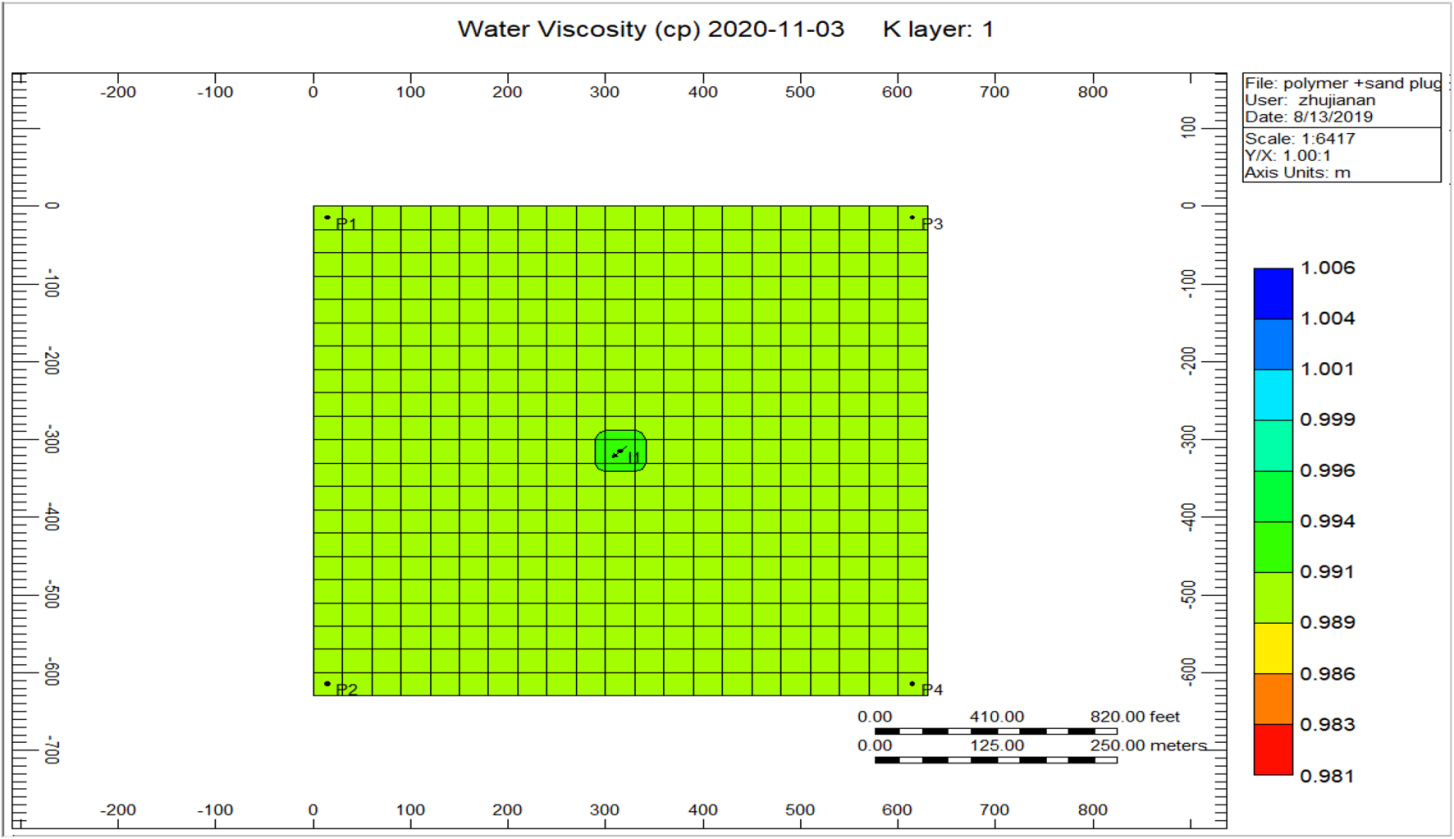
Shows the water viscosity of the cross-linked gel achievement model *CMG STARS 2017.10* URL: https://www.cmgl.ca/.

Figure 14 shows the water viscosity of the cross-linked gel achievement model, the viscosity of the water has augmented alongside an extent of 0.994 nearby the injector well along with a scope of 0.991 until 2020-11-03. Comparing to Fig. 8 shows the water viscosity during the polymer sand plug model is 0.981 because of the plugging effect taking place throughout polymer flooding process, an accumulation of solid sand or a piece of movable sand toward the production well influencing the liquid rate together with the oil ratio to lessen based on the time that the sand–solid accumulate in the direction of the production well.

**Figure 15:**
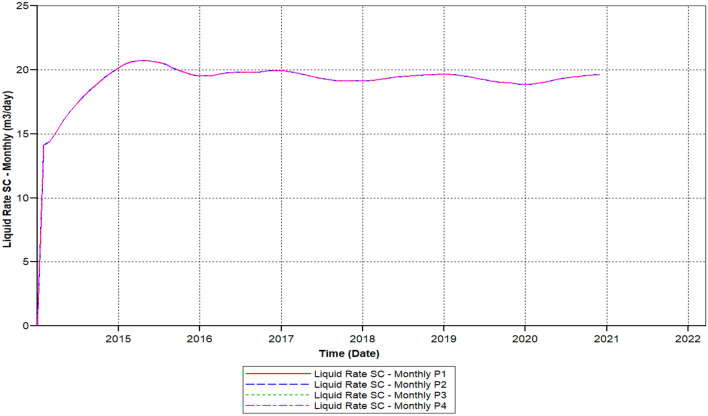
Shows the liquid ratio afterwards the cross-linked gel treatment graph *CMG STARS 2017.10* URL: https://www.cmgl.ca/.

**Figure 16:**
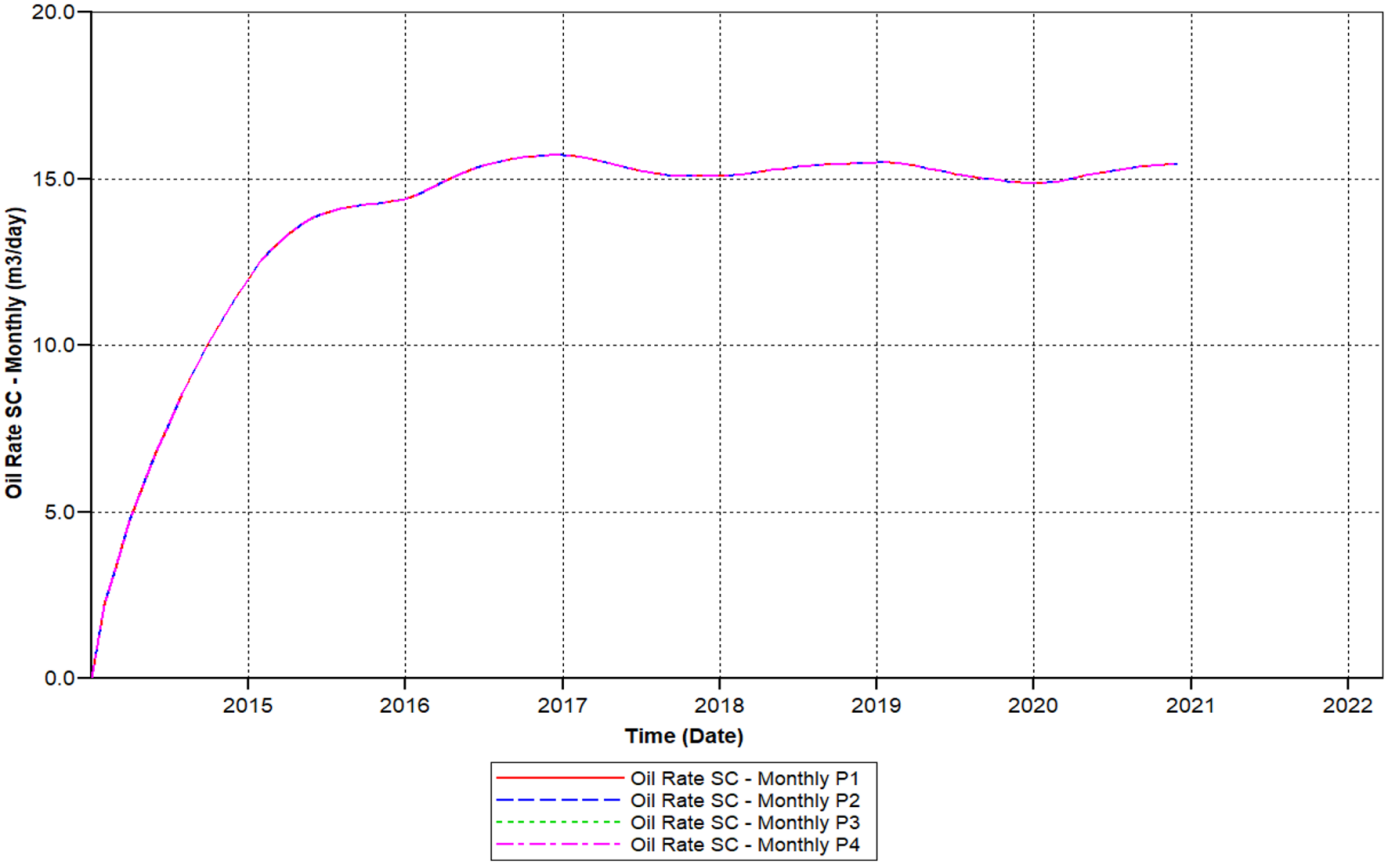
Shows the oil ratio afterwards the cross-linked gel treatment graph *CMG STARS 2017.10* URL: https://www.cmgl.ca/.

We can observe the results after the usage of cross-linked gel treatment, plugging impact has been decreased and results have been ameliorated. Established on the chart outcome of; Fig. 15 indicates the liquid rate afterwards the cross-linked gel treatment graph including Fig. 16 shows the oil ratio afterwards the cross-linked gel treatment graph, cross-linked gel accomplished satisfactory outcomes; the blockage has crucially reduced on the liquid ratio and the oil rate can be observed on the graphs curve. This signifies, with the application of cross-linked gel achievement the affected variables can also be ameliorated. The implementation of cross-linked gel give shear solidity, warrant thermal solidity, erosion privilege for considering it is a strong gel interconnection.

Figure 15 shows the liquid ratio afterwards the cross-linked gel treatment graph, we can notice on the outcome that the liquid ratio commenced raising through the year; 2014-02-01 prior to it has outreached the higher ratio of 21.609 m^3^/day in 2015-01-02, then declined to the smallest range of 19.52 m^3^/day in 2020-02-03, without attaining 1 month the ratio started to rise again however it did not reached to 20 m^3^/day.

Figure 16 shows the oil ratio afterwards the cross-linked gel treatment graph commenced rising it has outreached a ratio about 14.47 m^3^/day in the year 2015-09-01 then the oil ratio declined to 15.214 m^3^/day within 2017-07-03 to 2018-01-02.

Even with the decrease, the ratio raised again beginning from the year 2018-02-04 to 2019-01-03 attained 15.48 m^3^/day. Throughout 2020-02-03, this ratio declined to a lower ratio of 14.93 m^3^/day. Eventually, the ratio has augmented to 15.32 m^3^/day for the remaining of the model simulation period.

### Sensitivity analysis

Along with the side effect on liquid ratio diminish and oil ratio diminishing unceasingly will be noticed throughout polymer flooding of polymer sand plug model along with cross-linked gel achievement model were contrasted with one another as detailed beneath;

#### Case 1: polymer sand plug model

We have lessened the initial solid concentration volume rate of 0.0521 of the starting model to a lower concentration rate of 0.023, plus the activation of energy element reaction frequency factor diminished to (FREQFACT = 0.00231211) for the reason of the steady decline of the liquid ratio together with oil ratio of the early polymer sand plug model (see Figs. 9, 10).

**Figure 17:**
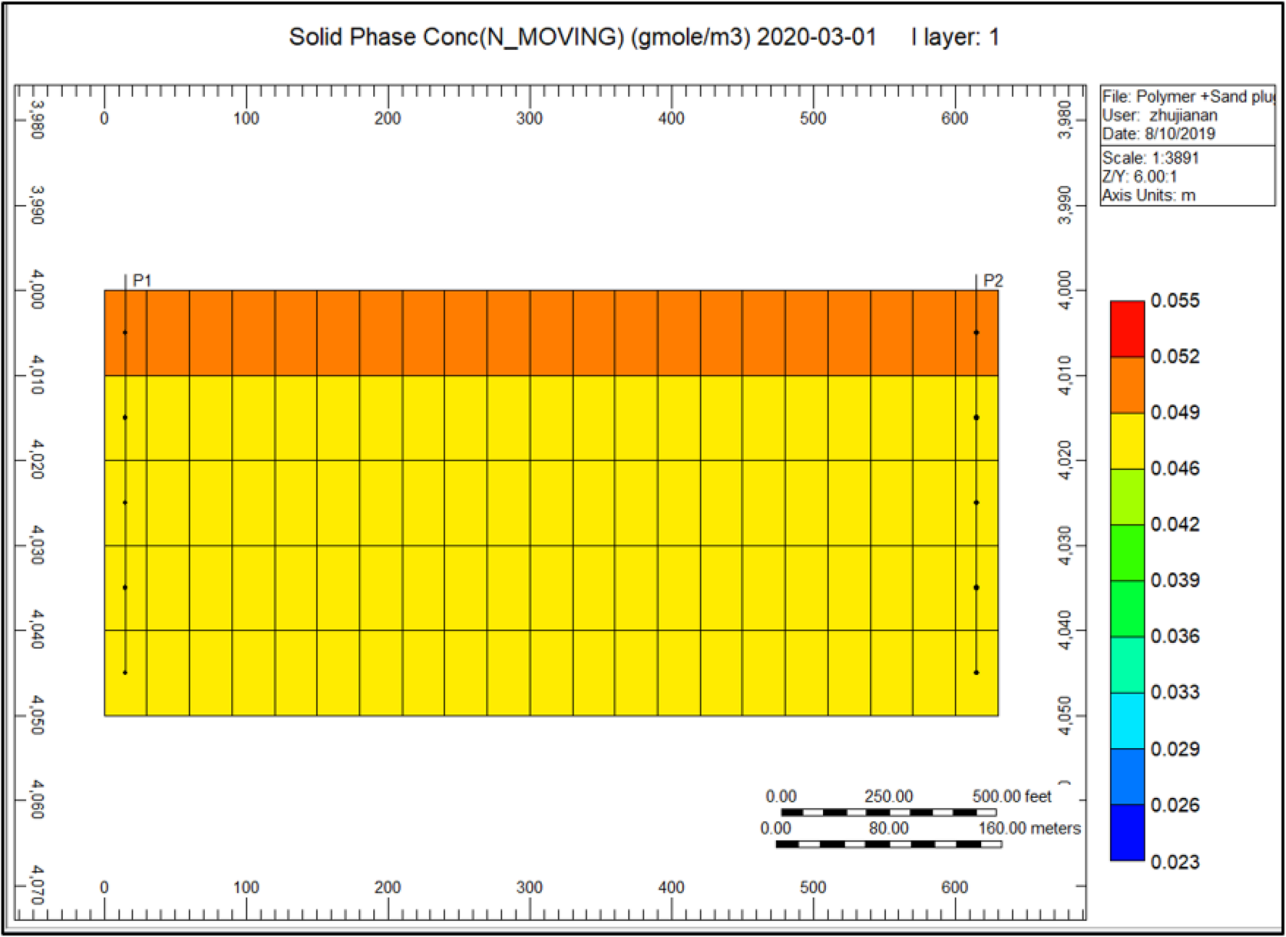
Indicates the solid-phase accumulation pointing to the production well *CMG STARS 2017.10* URL: https://www.cmgl.ca/.

**Figure 18:**
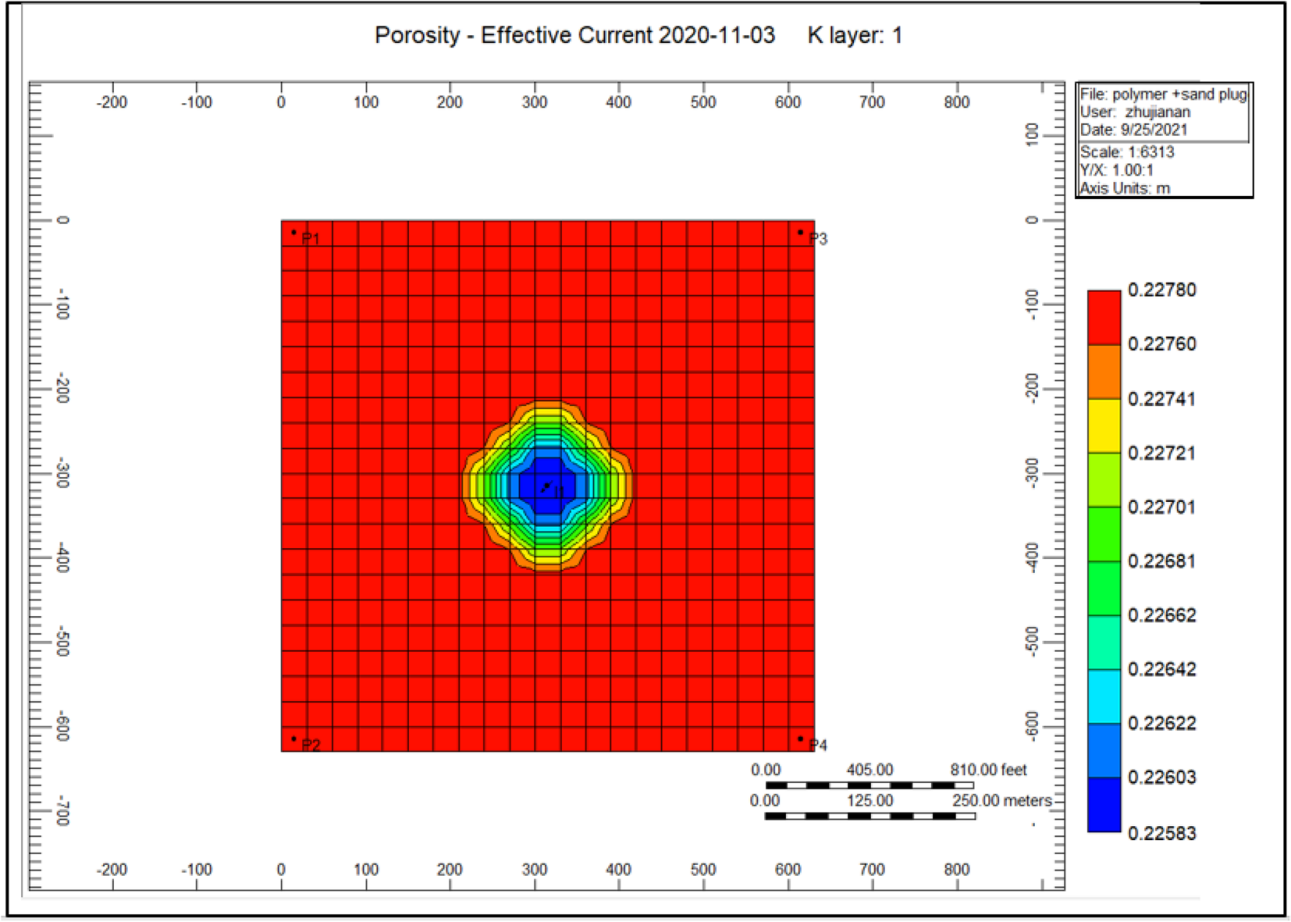
Shows the porosity diminish throughout the polymer sand plug model *CMG STARS 2017.10* URL: https://www.cmgl.ca/.

**Figure 19:**
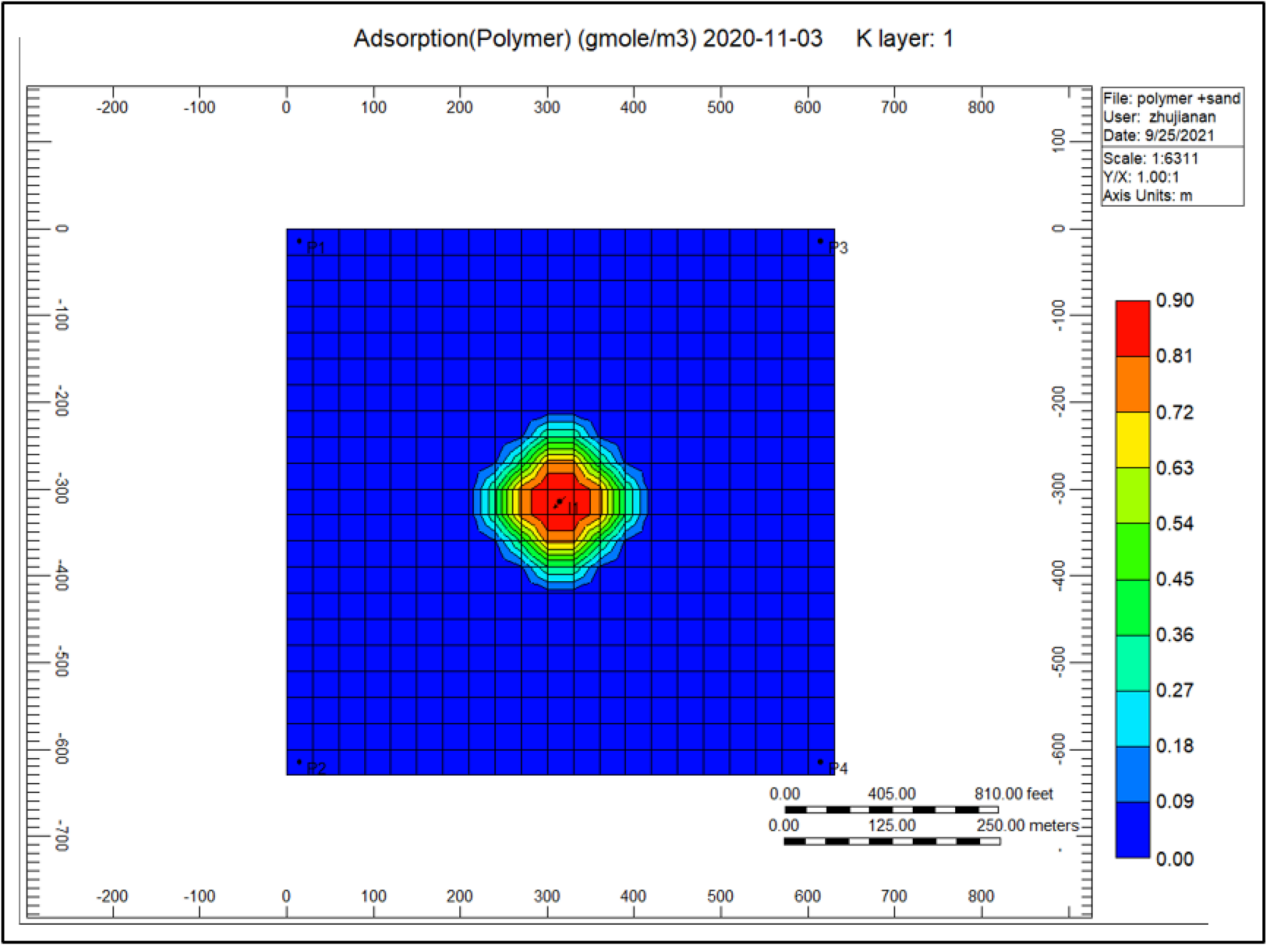
Indicates the polymer adsorption through the polymer sand plug model *CMG STARS 2017.10* URL: https://www.cmgl.ca/.

**Figure 20:**
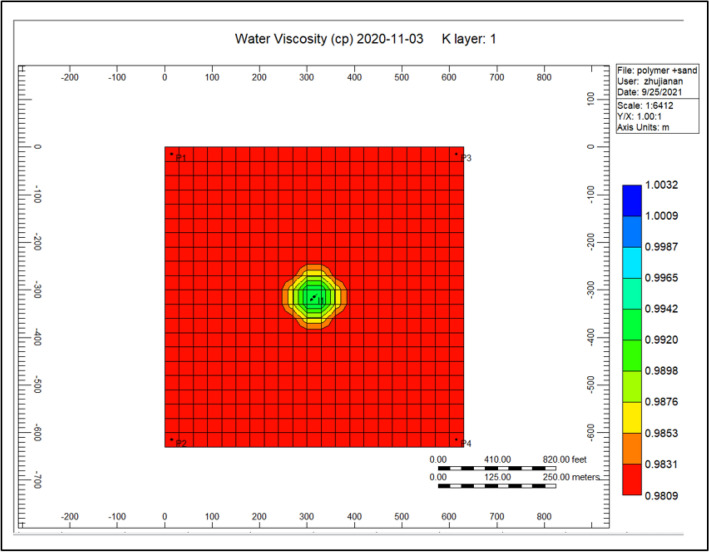
Shows the water viscosity of the polymer sand plug model CMG STARS 2017.10 URL: https://www.cmgl.ca/.

**Figure 21:**
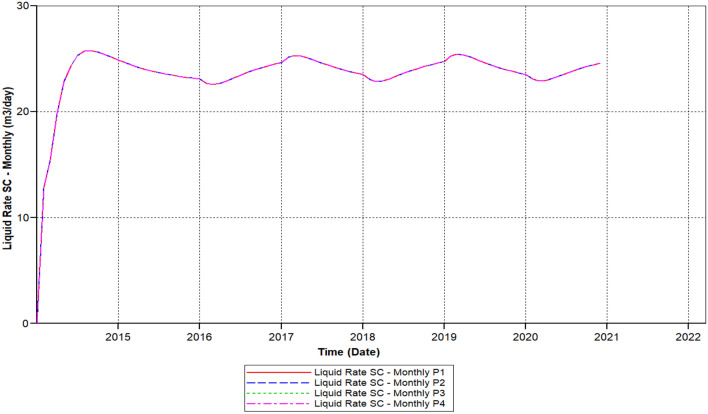
Shows the polymer sand plug model liquid ratio *CMG STARS 2017.10* URL: https://www.cmgl.ca/.

**Figure 22:**
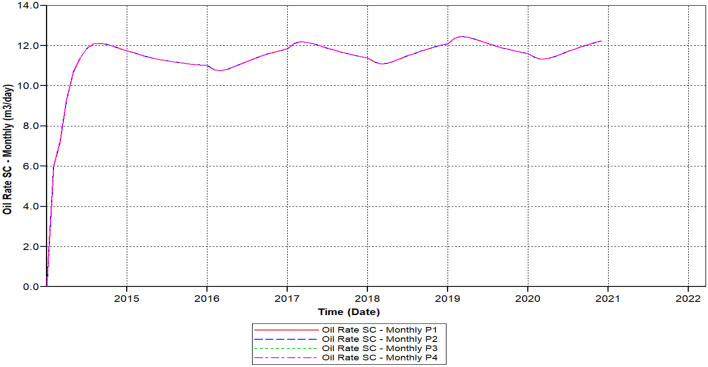
Shows the polymer sand plug model oil ratio *CMG STARS 2017.10* URL: https://www.cmgl.ca/.

We can notice with the decrease of solid-phase accumulation, the element reaction frequency factor reduces, and some of the affected parameters due to plugging have also improved. That improvement can be observed on the following figures; Fig. 17 indicates the accumulation of sand neighbouring the production well, Fig. 18 shows the porosity decrease through the polymer sand plug model, Fig. 19 indicates the polymer adsorption throughout the polymer sand plug model, Fig. 20 shows the water viscosity of the polymer sand plug model. We can notice on the base case model graph curves (see Fig. 9 shows the polymer sand plug model liquid ratio, Fig. 10 shows the polymer sand plug model oil ratio) the rate has experienced a continuous decrease. The liquid ratio and the oil ratio were afflicted before the lessening caused by the corking influence as we can notice on the curve graphs of; Fig. 21 shows the polymer sand plug model liquid ratio, Fig. 22 shows the polymer sand plug model oil ratio.

Figure 17 indicates the solid-phase accumulation pointing to the production well. We can view that the accumulation of solid-phase at the primary layer were accompanied with the scope of 0.049, whereas the remaining layers ranged to 0.046.

Figure 18 shows the porosity diminish throughout the polymer sand plug model, the porosity scope toward the injector well is 0.22563, while surrounding the producer well ranges to 0.22780.

Figure 19 indicates the polymer adsorption through polymer sand plug model, polymer adsorption is tremendously soaked up throughout the injector well with a range of 0.90, whereas nearby the producer well is nearly negligible.

Figure 20 shows the water viscosity of the polymer sand plug model, the viscosity of water nearby the injector well ranges 0.9853, while surrounding the producing well ranging to 0.9809.

Figure 21 shows the polymer sand plug model liquid ratio, the liquid ratio has attained a higher ratio of 25.810 m^3^/day in 2014-08-02, the rate commenced slowly decreasing and increasing from time to time, in the year 2018-03-03 reached a ratio of 23.452 m^3^/day. Afterwards, the liquid ratio steadily commenced rising has outreached a ratio of 24.74 m^3^/day in 2019-04-02, steadily the ratio commenced descending has outreached to a lower rate in the year 2020-03-03 to 23.853 m^3^/day. Furthermore, we can notice upon the graph that the ratio commenced rising and stable until the year 2020-12-1.

Figure 22 shows the polymer sand plug model oil ratio, whereas the oil ratio attained on it is foremost to 12.23 m^3^/day in 2014-08-04 also declined to 11.972 m^3^/day in the year 2014-12-01, we can see that in the year 2014 the oil ratio does not outreach to an elevated rate for the reason of the rapid increase along with sharp drop. The augmented ratio of 12.43 m^3^/day in 2017-04-02, throughout two months the oil ratio commenced lessening attained a ratio of 11.863 m^3^/day in the year 2018-03-02. Out of the diminish, the oil ratio begun recuperating till it has fulfilled an elevated rate contrasted to the preceding years, a ratio ranging to 12.60 m^3^/day accomplished in the year 2019-04-02. The rate decreased for a moment but started recovering for the rest of the simulation.

#### Case 2: cross-linked gel model

The solid-phase concentration adjusted comparable to the polymer sand plug model of case 1 ranges to 0.0231, this time the frequency reaction factor range minimized to 0.0000021. The bottommost frequency factor performs a preferable outcome on the cross-linked gel performance model base case.

**Figure 23:**
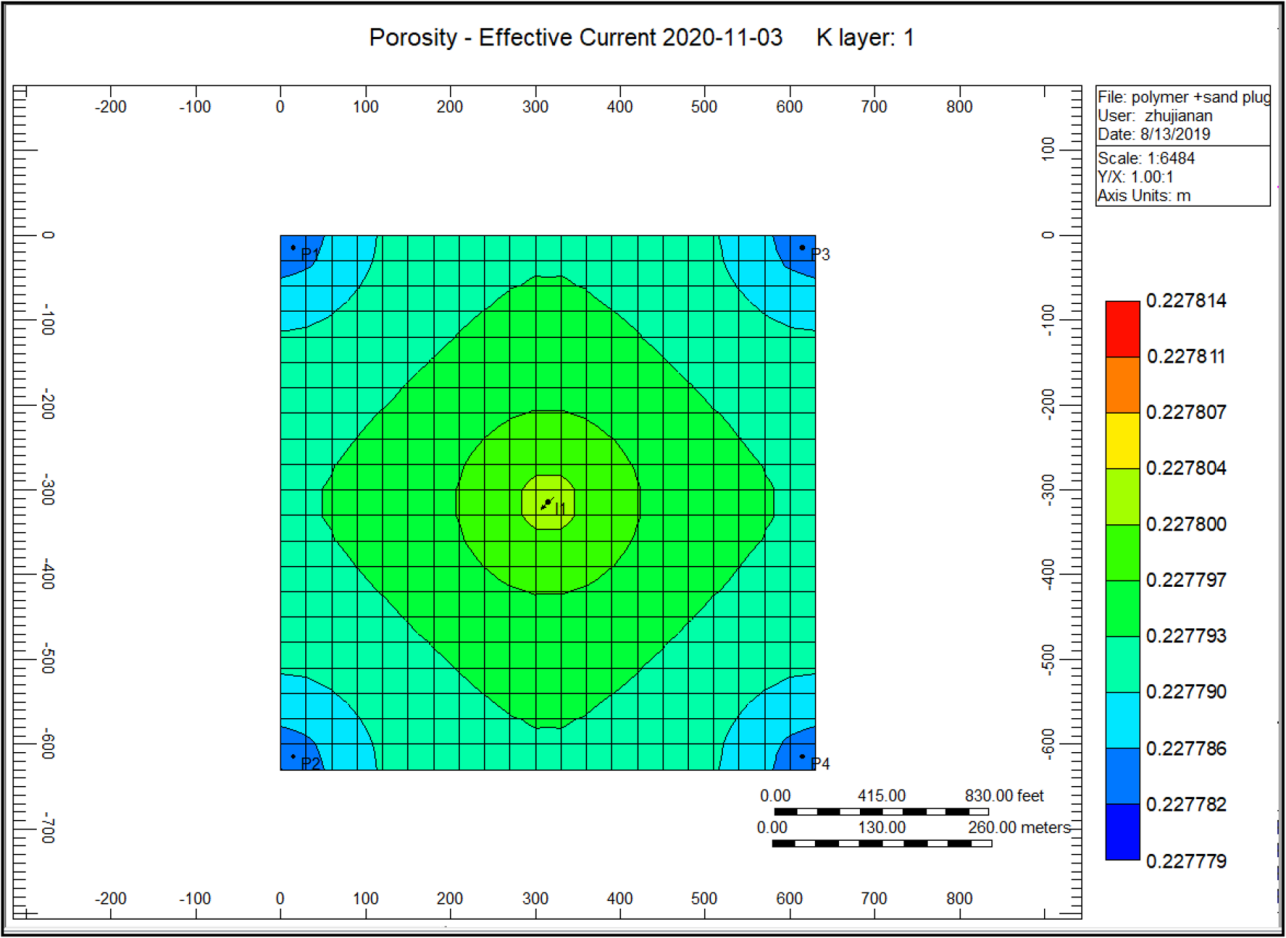
Indicates the porosity increased during the cross-linked gel performance 2020-11-03 *CMG STARS 2017.10* URL: https://www.cmgl.ca/.

Figure 23 indicates the porosity increased during the cross-linked gel performance 2020-11-03, the increment of porosity surrounding the injection well ranging to 0.227799, while the porosity ranges to 0.227784 in the direction of the rest of the model.

**Figure 24:**
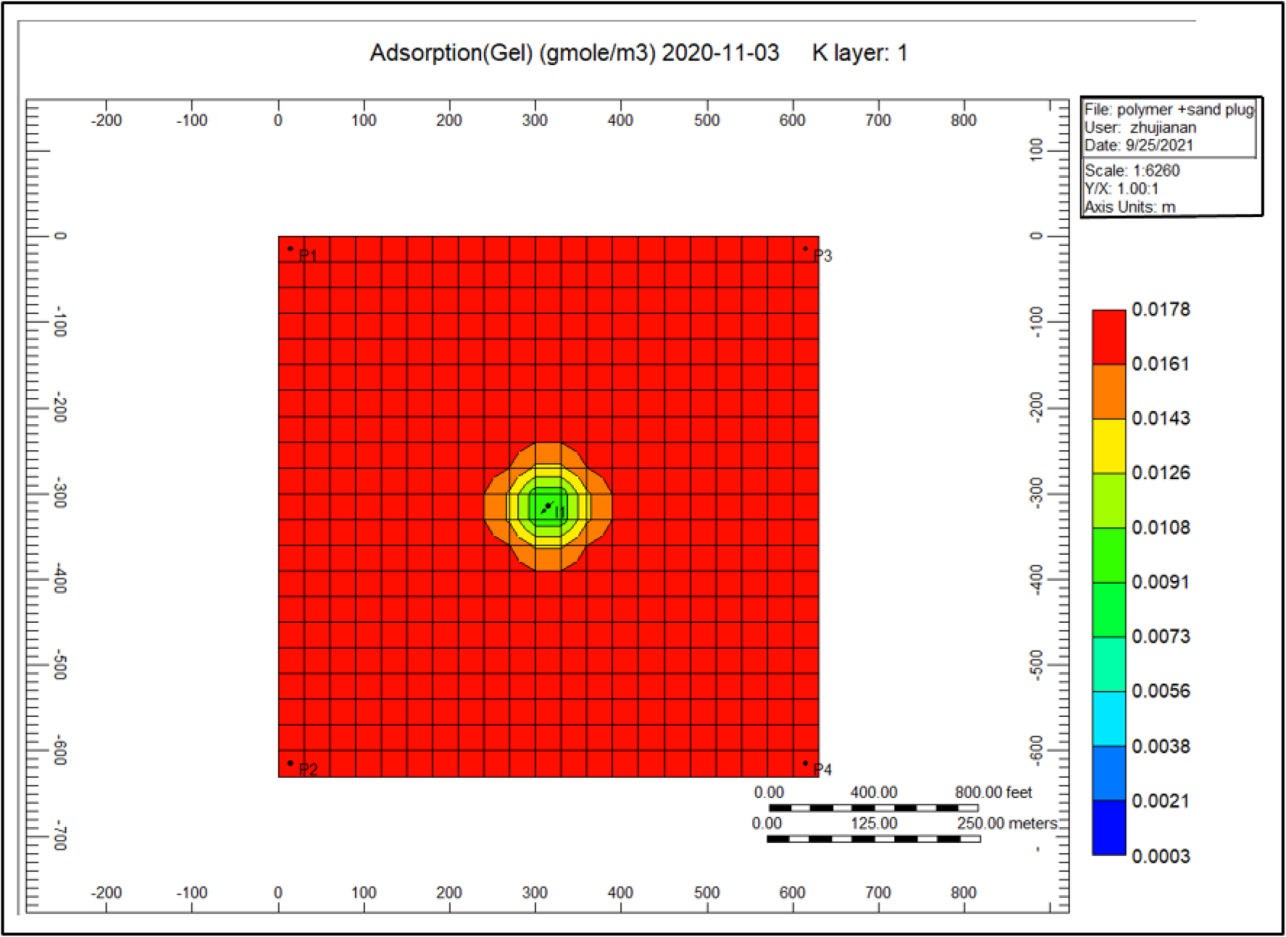
Shows the gel adsorption of the cross-linked gel achievement model 2020-11-03 *CMG STARS 2017.10* URL: https://www.cmgl.ca/.

Figure 24 shows the gel adsorption of the cross-linked achievement model 2020-11-03, gel cross-linked is thoroughly soaked up pointing to the whole model ranges to 0.0178, whereas nearby the injector well the adsorption ranging to 0.013.

**Figure 25:**
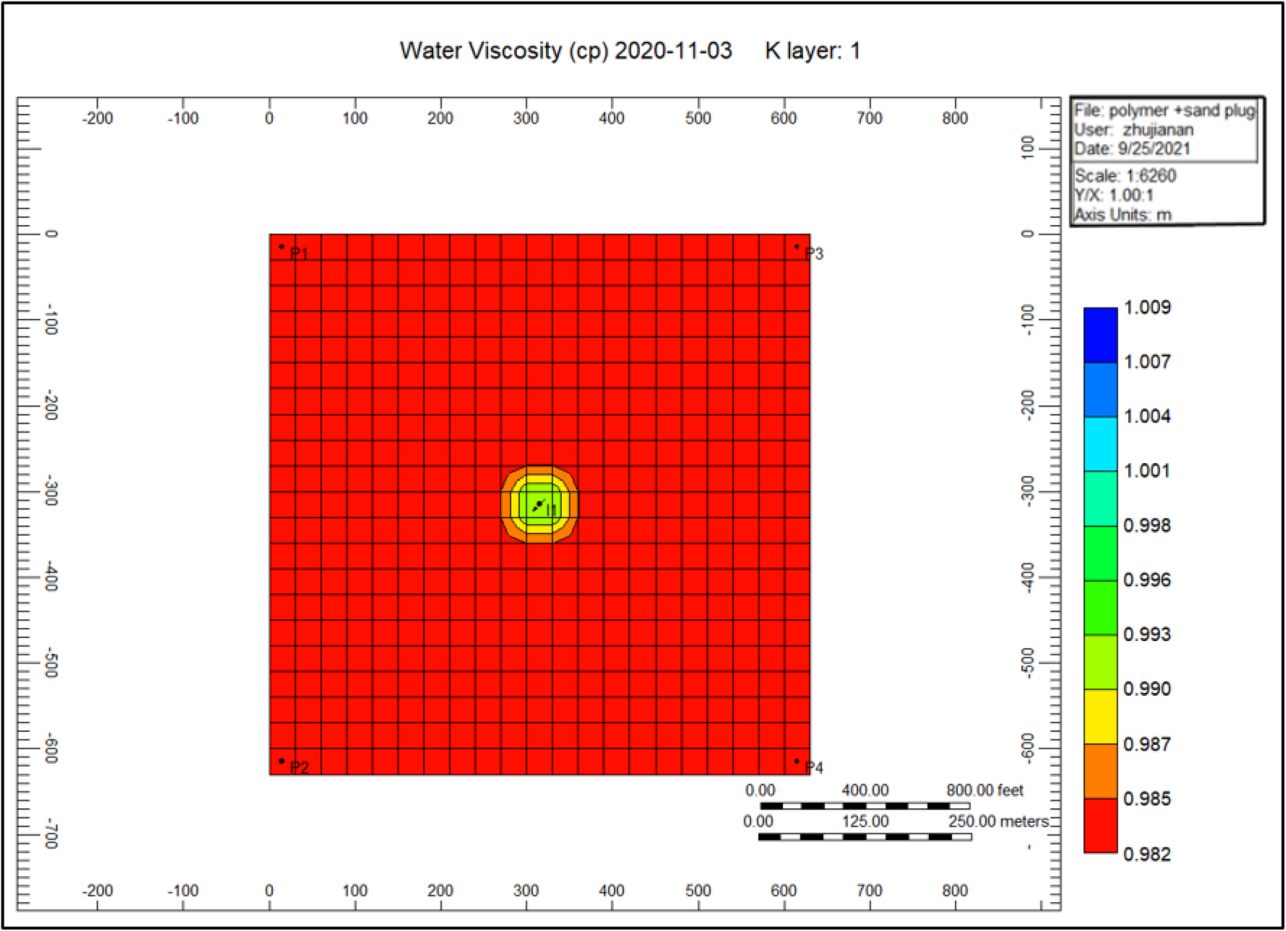
Shows the water viscosity of the cross-linked gel achievement 2020-11-03 *CMG STARS 2017.10* URL: https://www.cmgl.ca/.

Figure 25 shows the water viscosity of the cross-linked gel achievement 2020-11-03, we can remark on the figure that the viscosity ranges to 0.987 surrounding the injector well, whereas the rest of the model ranges 0.982.

**Figure 26:**
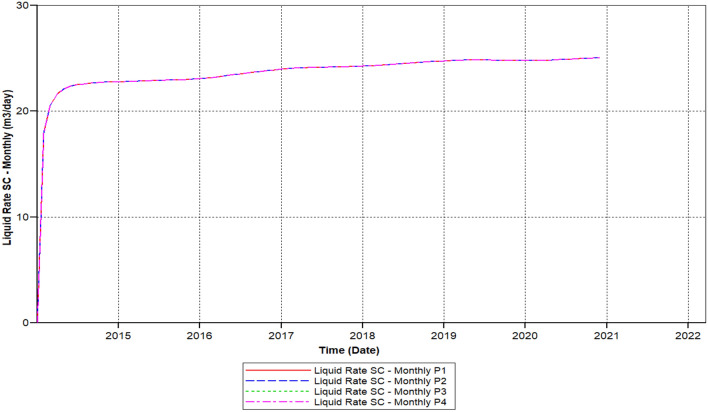
Indicates the liquid ratio afterward the cross-linked gel treatment graph *CMG STARS 2017.10* URL: https://www.cmgl.ca/.

**Figure 27:**
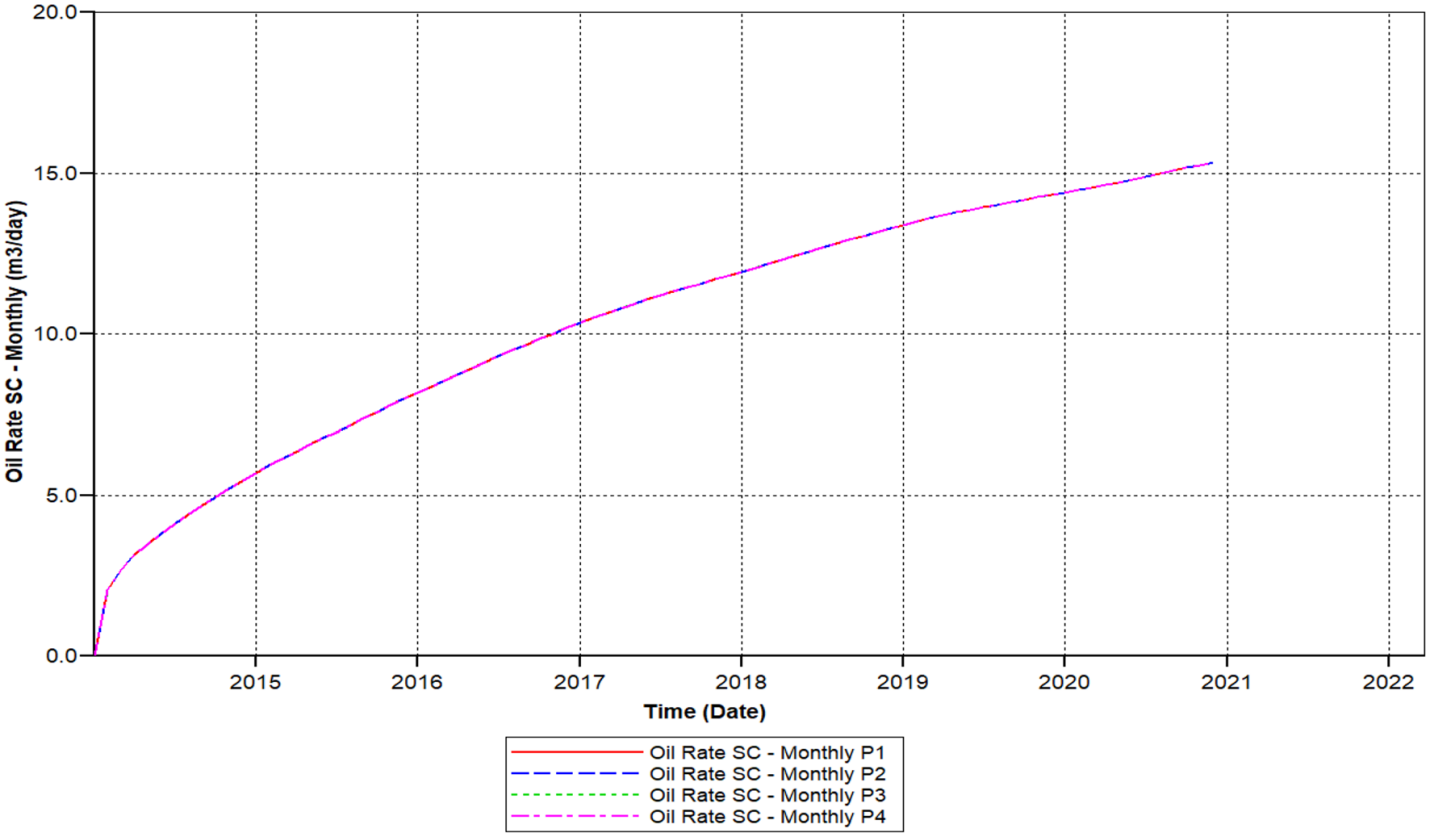
Shows the oil ratio afterwards the cross-linked gel treatment graph *CMG STARS 2017.10* URL: https://www.cmgl.ca/.

We can notice based on the outcome evaluation that, with the reduction of solid-phase accumulation, frequency reaction factor diminution has lessened ratio contrasted to the prior Fig. 15 indicates the liquid rate afterwards the cross-linked gel treatment graph, while the ongoing Fig. 26 indicates the liquid ratio succeeding the cross-linked gel treatment graph, achieved away preferable outcomes with nearly no presence of plugging impacts, alongside a more significant liquid ratio differentiating to Fig. 15, however the present graph, Fig. 27 shows the oil ratio following the cross-linked gel treatment graph, displays the plugging effect did not occurred at all upon the graph, except the oil ratio is smaller compare to Fig. 16 shows the oil ratio afterwards the cross-linked gel treatment graph.

Figure 26 indicates the liquid ratio afterwards the cross-linked gel treatment graph, with the decrease of solid-phase accumulation, along with the frequency reaction factor diminish we could remark through this graph that the liquid rate importantly improving without encountering any liquid ratio reduction. With a commencing ratio of 23.52 m^3^/day in the year 2014-03-02 toward 2015-10-03, ceaselessly rising free from ratio decline attained a rate of 24.54 m^3^/day in 2017-01-02. Progressively the liquid ratio rising, within the year 2019-01-03 attained the elevated ratio of 25.43 m^3^/day, the liquid ratio steady up to 2020-12-02.

Figure 27 shows the oil ratio afterwards the cross-linked gel treatment graph, along with bottommost solid-phase accumulation and frequency reaction factor, oil ratio initiated rising unceasingly free from encountering somewhat a decline through the year 2014-01-01 up to it has outreached it is elevated ratio 15.22 m^3^/day in 2020-12-02.

The finding of the previous studies mentioned that the productivity losses in polymer floods have been assigned to numerous factors and its feasible amalgamation:

- Skin damage nearby the wellbore (i.e. asphaltene deposition, mud formation, etc.)
- Plugging outcomes because of the existence of solid fine fragments.
- Decreased fluid removal in artificial lift pump structure because of expansions in fluid density in the existence of produced polymer.
- Stable oil–water–polymer emulsions.

Another finding mentioned that; solid-phase invasion, fines migration, scale formation, as well as water sensitivity can occasion pollution and plugging nearby the wellbore; therefore has an unfavourable impact on oil production.

This researcher proposed a acidizing treatment is one of the extremely crucial techniques that used in aiming to bring out the pollution and recover formation permeability.

An insight was generated through our research study results on the base cases (polymer flood model, cross-linked gel model) furthermore on sensitivity cases (polymer flood model, cross-linked gel model) that the liquid ratio, oil ratio of polymer model diminishes faster furthermore unsteady apart from the cross-linked gel model indicate stability.

The bigger the SOLIDMIN and the FREQFAC, the more terrible the liquid ratio, oil ratio were remarked on the base case (polymer flood model).

The foremost parameters of the plugging mechanism who was engendering production liquid decrease investigated plus ameliorated as shown:

1. Solid-phase concentration toward the wells and FREQFACT are the dominant influences originating plugging also directing the production rate to diminish.
2. Throughout polymer flooding, the liquid rate, oil rate lessened because of the plugging mechanism, the application of cross-linked gel boosted reservoir production rate.
3. Along with the approach of polymer flooding, the viscosity has enlarged accompanied by the plugging effect, however, water viscosity diminished, enhanced the displacement and, also the application of the cross-linked gel.
4. Comparing with polymer flood model based case and polymer flood model case 1, the cross-linked gel model of Case 2 performed a far better result with no plugging occurring and production rate increased, followed by the base case cross-linked gel model the plugging impact is decreased.
5. For a better understanding of the adsorption of polymer, it was highly recommended to study and investigate the adsorption of polymer on the surface of the core or sandstone powder (reservoir rock) for further research.

## Conclusion

Based upon the outcomes, it can be concluded that the parameters affecting the reservoir were stated as below:

1. The parameters influencing the liquid rate, oil rate to decline given the existence of sand fragments, junks who were generating plugging throughout polymer flooding as shown: Polymer adsorption, polymer deterioration whichever influences the permeability to diminish, porosity reduction, inaccessible pore volume, water viscosity, the elevated accumulation of solid-phase along with the elevated reactant frequency factor along with an elevated ratio could impact poorly the liquid ratio, oil ratio to diminish speedily on the other hand steadily derived from the specification of the parameters (reactant frequency factor along with the accumulation of solid-phase) as well as will direct to production deficit.
2. As result of the sand plugging circumstance taking place throughout the polymer flooding influencing the liquid ratio also oil ratio to diminish unceasingly, exploring for preventive treatment to lessen polymer plugging moreover ameliorating the liquid with oil ratio was exceptionally demanded.
3. We could remark on the outcome after the implementation of cross-linked gel treatment on the polymer sand plugged model impact were decreased and the outcome was enhanced.
4. Cross-linked gel soaking up, permeability, porosity, water viscosity, the accumulation of solid-phase were boosted also the production rate were improved.
5. With the application of cross-linked gel treatment fulfilled a satisfactory outcomes; the solid-phase concentration volume and the plugging have been remarkably diminished on the liquid ratio as well as oil ratio though we can observe some decline.
6. As the solid-phase concentration volume and the reactant frequency factor rate decreased in the polymer plug model Case 1 achieved better results compare to polymer plug model base case with less concentration of solid-phase, plugging and better production rate.
7. Comparing with polymer flood model based case and polymer flood model case 1, the cross-linked gel model of Case 2 performed a far better result with no plugging occurring and production rate increased, followed by the base case cross-linked gel model the plugging impact is decreased.
8. Comparing with polymer flood model based case and polymer flood model case 1, the cross-linked gel model of Case 2 performed a far better result with no plugging occurring and production rate increased, followed by the base case cross-linked gel model the plugging impact has been decreased.
9. The implementation of cross-linked gel affords shear solidity, promising thermal reliability, erosion release due to it is a robust gel mesh.
10. Our research results found the main parameters which are causing the productivity losses and decreased the plugging effects by the cross-linked gel treatment.
11. Future work has to be concentrated more on understanding and improving the parameters of (SOLIDMIN) and (FREQFAC) in order to enhance the reservoir performance.

## Data Availability

The data initiated throughout or examined through the ongoing study are available from the corresponding author upon request.
